# Online citizen sciences reveal natural enemies and new occurrence data of *Meteorusstellatus* Fujie, Shimizu & Maeto, 2021 (Hymenoptera, Braconidae, Euphorinae)

**DOI:** 10.3897/BDJ.11.e103436

**Published:** 2023-04-19

**Authors:** So Shimizu, Hsuan-Pu Chen, Kai-Ti Lin, Ren-Jye Chen, Shunpei Fujie, Su-Chuan Hung, Mei-Ling Lo, Ke-Hsiung Tsai, Kaoru Maeto

**Affiliations:** 1 Institute for Agro-Environmental Sciences, NARO, Tsukuba, Japan Institute for Agro-Environmental Sciences, NARO Tsukuba Japan; 2 Laboratory of Insect Biodiversity and Ecosystem Science, Graduate School of Agricultural Science, Kobe University, Kobe, Japan Laboratory of Insect Biodiversity and Ecosystem Science, Graduate School of Agricultural Science, Kobe University Kobe Japan; 3 Department of Entomology, National Taiwan University, Taipei, Taiwan Department of Entomology, National Taiwan University Taipei Taiwan; 4 Kaohsiung Association of Naturalists, Kaohsiung, Taiwan Kaohsiung Association of Naturalists Kaohsiung Taiwan; 5 Osaka Museum of Natural History, Osaka, Japan Osaka Museum of Natural History Osaka Japan; 6 Zhongzheng Community College, Taipei, Taiwan Zhongzheng Community College Taipei Taiwan; 7 Butterfly Conservation Society of Taiwan, Taipei, Taiwan Butterfly Conservation Society of Taiwan Taipei Taiwan; 8 The Society of Wilderness, Taoyuan Branch, Taoyuan, Taiwan The Society of Wilderness, Taoyuan Branch Taoyuan Taiwan; 9 Wild Bird Society of Taoyuan, Taoyuan, Taiwan Wild Bird Society of Taoyuan Taoyuan Taiwan

**Keywords:** Facebook, Ichneumonidae, Lepidoptera, parasitoid wasp, predator, Pteromalidae, social media, Sphingidae, Trichogrammatidae, Vespidae

## Abstract

**Background:**

Citizen science is a research approach that involves collaboration between professional scientists and non-professional volunteers. The utilisation of recent online citizen-science platforms (e.g. social networking services) has greatly revolutionised the accessibility of biodiversity data by providing opportunities for connecting professional and citizen scientists worldwide. *Meteorusstellatus* Fujie, Shimizu & Maeto, 2021 (Hymenoptera, Braconidae, Euphorinae) has been recorded from the Oriental Islands of Japan and known to be a gregarious endoparasitoid of two macro-sized sphingid moths of *Macroglossum*, *Ma.passalus* (Drury) and *Ma.pyrrhosticta* Butler. It constructs characteristic star-shaped communal cocoons, suspended by a long cable. Although *M.stellatus* has been reported only from the Oriental Islands of Japan, the authors recognise its occurrence and ecological data from Taiwan and the Palaearctic Island of Japan through posts on online citizen-science groups about Taiwanese Insects on Facebook and an article on a Japanese citizen-scientist's website.

**New information:**

Through collaboration between professional and citizen scientists via social media (Facebook groups) and websites, the following new biodiversity and ecological data associated with *M.stellatus* are provided:

1. *Meteorusstellatus* is recorded for the first time from Taiwan and the Palaearctic Region (Yakushima Is., Japan).
2. *Cechetraminor* (Butler, 1875), *Hippotioncelerio* (Linnaeus, 1758) and *Macroglossumsitiene* (Walker, 1856) (Lepidoptera, Sphingidae) are recorded for the first time as hosts of *M.stellatus* and two of which (*C.minor* and *H.celerio*) represent the first genus-level host records for *M.stellatus*.
3. *Mesochorus* sp. (Hymenoptera, Ichneumonidae), indeterminate species of Pteromalidae and Trichogrammatidae (Hymenoptera), are recognised as hyperparasitoid wasps of *M.stellatus*.
4. *Parapolybiavaria* (Fabricius, 1787) (Hymenoptera, Vespidae) is reported as a predator of pendulous communal cocoons of *M.stellatus*.

The nature of suspended large-sized communal cocoons of *M.stellatus* and the importance and limitations of digital occurrence data and online citizen science are briefly discussed.

## Introduction

Citizen science is a research approach that involves collaboration between professional scientists and non-professional volunteers, aimed at enhancing the ability of scientific data collection and expanding its purview to scales or resolutions beyond the capabilities of individual researchers or research teams and has made significant contributions to science, education and society (e.g. Cohn (2008), Bonney et al. (2009), Silvertown (2009), Newman et al. (2012), Bonney et al. (2014), Kobori et al. (2016)). The recent rapid advancements in internet technology have considerably facilitated the active implementation of larger-scale online citizen-science projects across various fields of science (e.g. Dickinson et al. (2012), Kobori et al. (2016), Chandler et al. (2017), Kyba et al. (2023)). These projects utilise online platforms, such as social networking services (SNS) (e.g. Facebook, Instagram and Twitter) and specialised citizen-science applications (e.g. iNaturalist), surpassing previous citizen science in scope and magnitude.

For only professional scientists, it would be hardly possible to obtain comprehensive biodiversity data of hyper-diverse life on Earth. Biodiversity studies have, therefore, traditionally been supported by not only professional researchers, but also amateur citizen scientists. Natural history museums historically play a central role in supporting amateur scientists and developing citizen science up to date (Sforzi et al. 2018), but the communities on online platforms recently frequently take a similar role. The utilisation of online citizen-science platforms has greatly revolutionised the accessibility of biodiversity data by providing opportunities for connecting professional and citizen scientists worldwide. As a result, many new important discoveries and data have been published (e.g. Gonella et al. (2015), Jaume-Schinkel et al. (2020), Santamaria et al. (2020), Zhang et al. (2022)).

*Meteorusstellatus* Fujie, Shimizu & Maeto, 2021 is a recently described braconid parasitoid wasp species of the *M.pulchricornis* clade from the Oriental Region, Ryukyu Islands, Japan (Fujie et al. 2021). It has been known to be a gregarious endoparasitoid of two macro-sized sphingid moths of *Macroglossum*, *Ma.passalus* (Drury) and *Ma.pyrrhosticta* Butler (Fujie et al. 2021). One of the most interesting features of *M.stellatus* is its characteristic star-shaped communal cocoons, suspended by a long cable. Fujie et al. (2021) suggested that this unique cocoon morphology likely contributes to reducing the risk of hyperparasitism by minimising the exposed area of each individual cocoon as observed in the microgastrine gregarious braconid parasitoid *Cotesiaglomerata* (Linnaeus) (Tagawa and Fukushima 1993, Tanaka and Ohsaki 2006).

Although *M.stellatus* has been reported only from the Oriental Islands of Japan, the authors recognise its occurrence and ecological data from Taiwan and the Palaearctic Island of Japan through posts on online citizen-science groups about Taiwanese Insects on Facebook and an article on a Japanese citizen-scientist's website. Therefore, the authors conducted an online citizen science-based investigation on *M.stellatus* via social media and the present paper aims to record *M.stellatus* from these regions for the first time, to report some new ecological data associated with it and to re-evaluate the function of the characteristic cocoon.

## Materials and methods

### Online data compilation

All digital occurrence data of *M.stellatus* were manually compiled from three online citizen-science groups of Taiwanese insects on Facebook (Table 1) and an article on a Japanese citizen scientist's website without automated web crawler programmes. The data were searched by specific keywords, such as "懸繭蜂" (the common Chinese name for the genus *Meteorus*) and "*Meteorus*." We tried to contact all the posters to obtain permission to reuse and edit their digital occurrence data of *M.stellatus*, including the original resolution photographs and to gather additional relevant information through communication with them. The data which failed to obtain a poster's response to our permission offer were excluded from our results in accordance with ethical considerations. All the obtained digital occurrence data were shown as figures and summarised as tables in the present paper since the original posts and links are probably not permanent.

### Identification of digital occurrence data

The compiled digital occurrence data of *M.stellatus* and related insects (i.e. hosts, hyperparasitoids and predators) were initially identified, based on images or movies. Subsequently, identification was confirmed by morphological observation of specimens using a stereoscopic microscope (SMZ1500, Nikon, Tokyo, Japan) if the original samples were available. Examined specimens were mounted and preserved in public institutions (Table 2).

### Figure editing

The habitus of *M.stellatus* was newly photographed for the present paper with the technique described by Shimizu and Broad (2020) and Shimizu et al. (2020). Original photographs of digital occurrence data were provided by each poster and developed and edited using Adobe Illustrator 2023 and Photoshop 2023 (Adobe Systems Inc., San Jose, CA, USA). The distribution map was made by QGIS v.3.28.1 (QGIS.org 2023).

## Taxon treatments

### Meteorus stellatus

Fujie, Shimizu & Maeto, 2021

BF184A91-11EA-518C-9155-07B37BA5F29E

D8785F79-E874-4854-95D7-5C0A928914CA

*Meteorusstellatus* Fujie, Shimizu & Maeto, 2021: 27; holotype ♀ from Japan, deposited at Osaka Museum of Natural History, Osaka, Japan (OMNH).

#### Materials

- **Type status:** Other material. **Occurrence:** occurrenceRemarks: Communal wasp cocoons and its host larva (*Cechetraminor*) were observed.; recordedBy: Mei-Ling Lo; lifeStage: cocoon; occurrenceID: 10BE637B-5C40-5090-A5BC-CB05ED4C2683; **Taxon:** taxonID: urn:lsid:zoobank.org:act:D8785F79-E874-4854-95D7-5C0A928914CA; scientificName: *Meteorusstellatus* Fujie, Shimizu & Maeto, 2021; acceptedNameUsage: *Meteorusstellatus* Fujie, Shimizu & Maeto, 2021; originalNameUsage: *Meteorusstellatus* Fujie, Shimizu & Maeto, 2021; kingdom: Animalia; phylum: Arthropoda; class: Insecta; order: Hymenoptera; family: Braconidae; genus: Meteorus; specificEpithet: *stellatus*; taxonRank: species; scientificNameAuthorship: Fujie, Shimizu & Maeto; vernacularName: ホシガタハラボソコマユバチ; nomenclaturalCode: ICZN; taxonomicStatus: accepted; **Location:** continent: Asia; islandGroup: Taiwan; island: Taiwan; country: Taiwan; countryCode: TW; stateProvince: Taoyuan; municipality: Fuxing District; locality: Xiaoyun Elementary School, Xiayun; **Identification:** identifiedBy: So Shimizu, Hsuan-Pu Chen, Kai-Ti Lin, Shunpei Fujie, Kaoru Maeto; dateIdentified: 2022; identificationReferences: Fujie et al. 2021; **Event:** eventDate: 14/07/2012; year: 2012; month: 7; day: 14; **Record Level:** type: StillImage; language: zh; basisOfRecord: HumanObservation; source: https://www.facebook.com/groups/588350308851792/permalink/627584148261741/
- **Type status:** Other material. **Occurrence:** occurrenceRemarks: Adult wasps were already emerged and cocoons were empty.; recordedBy: Shu-Ping Yang; lifeStage: emerged cocoon; occurrenceID: 8D1870CD-7746-598E-998D-F71CDED4F6BE; **Taxon:** taxonID: urn:lsid:zoobank.org:act:D8785F79-E874-4854-95D7-5C0A928914CA; scientificName: *Meteorusstellatus* Fujie, Shimizu & Maeto, 2021; acceptedNameUsage: *Meteorusstellatus* Fujie, Shimizu & Maeto, 2021; originalNameUsage: *Meteorusstellatus* Fujie, Shimizu & Maeto, 2021; kingdom: Animalia; phylum: Arthropoda; class: Insecta; order: Hymenoptera; family: Braconidae; genus: Meteorus; specificEpithet: *stellatus*; taxonRank: species; scientificNameAuthorship: Fujie, Shimizu & Maeto; vernacularName: ホシガタハラボソコマユバチ; nomenclaturalCode: ICZN; taxonomicStatus: accepted; **Location:** continent: Asia; islandGroup: Taiwan; island: Taiwan; country: Taiwan; countryCode: TW; stateProvince: Taipei; municipality: Shihlin District; locality: Mt. Dalunwei; **Identification:** identifiedBy: So Shimizu, Hsuan-Pu Chen, Kai-Ti Lin, Shunpei Fujie, Kaoru Maeto; dateIdentified: 2022; identificationReferences: Fujie et al. 2021; **Event:** eventDate: 24/08/2013; year: 2013; month: 8; day: 24; **Record Level:** type: StillImage; language: zh; basisOfRecord: HumanObservation; source: https://www.facebook.com/groups/369189783201470/permalink/423000581153723/
- **Type status:** Other material. **Occurrence:** occurrenceRemarks: Adult wasps were already emerged and cocoons were empty.; recordedBy: Tieh Hu (胡蝶); lifeStage: emerged cocoon; occurrenceID: A0001BE2-FC4F-52BE-8629-6B8A59CD9813; **Taxon:** taxonID: urn:lsid:zoobank.org:act:D8785F79-E874-4854-95D7-5C0A928914CA; scientificName: *Meteorusstellatus* Fujie, Shimizu & Maeto, 2021; acceptedNameUsage: *Meteorusstellatus* Fujie, Shimizu & Maeto, 2021; originalNameUsage: *Meteorusstellatus* Fujie, Shimizu & Maeto, 2021; kingdom: Animalia; phylum: Arthropoda; class: Insecta; order: Hymenoptera; family: Braconidae; genus: Meteorus; specificEpithet: *stellatus*; taxonRank: species; scientificNameAuthorship: Fujie, Shimizu & Maeto; vernacularName: ホシガタハラボソコマユバチ; nomenclaturalCode: ICZN; taxonomicStatus: accepted; **Location:** continent: Asia; islandGroup: Taiwan; island: Taiwan; country: Taiwan; countryCode: TW; stateProvince: Taipei; municipality: Beitou District; locality: Yangmingshan National Park; **Identification:** identifiedBy: So Shimizu, Hsuan-Pu Chen, Kai-Ti Lin, Shunpei Fujie, Kaoru Maeto; dateIdentified: 2022; identificationReferences: Fujie et al. 2021; **Event:** eventDate: 07/12/2013; year: 2013; month: 12; day: 7; **Record Level:** type: StillImage; language: zh; basisOfRecord: HumanObservation; source: https://www.facebook.com/groups/369189783201470/permalink/475675245886256/
- **Type status:** Other material. **Occurrence:** occurrenceRemarks: Wasp cocoon and its hyperparasitoid wasp (Trichogrammatidae sp.) were observed.; recordedBy: Mei-Ling Lo; lifeStage: cocoon; occurrenceID: FE859DC6-4FFD-5A04-87E3-4752D6880D2E; **Taxon:** taxonID: urn:lsid:zoobank.org:act:D8785F79-E874-4854-95D7-5C0A928914CA; scientificName: *Meteorusstellatus* Fujie, Shimizu & Maeto, 2021; acceptedNameUsage: *Meteorusstellatus* Fujie, Shimizu & Maeto, 2021; originalNameUsage: *Meteorusstellatus* Fujie, Shimizu & Maeto, 2021; kingdom: Animalia; phylum: Arthropoda; class: Insecta; order: Hymenoptera; family: Braconidae; genus: Meteorus; specificEpithet: *stellatus*; taxonRank: species; scientificNameAuthorship: Fujie, Shimizu & Maeto; vernacularName: ホシガタハラボソコマユバチ; nomenclaturalCode: ICZN; taxonomicStatus: accepted; **Location:** continent: Asia; islandGroup: Taiwan; island: Taiwan; country: Taiwan; countryCode: TW; stateProvince: Taoyuan; municipality: Guishan District; locality: Futoushan trail; **Identification:** identifiedBy: So Shimizu, Hsuan-Pu Chen, Kai-Ti Lin, Shunpei Fujie, Kaoru Maeto; dateIdentified: 2022; identificationReferences: Fujie et al. 2021; **Event:** eventDate: 23/12/2015; year: 2015; month: 12; day: 23; **Record Level:** type: StillImage; language: zh; basisOfRecord: HumanObservation; source: https://www.facebook.com/groups/393148477475231/permalink/802219766568098/
- **Type status:** Other material. **Occurrence:** occurrenceRemarks: Wasp cocoon and its hyperparasitoid wasp (Pteromalidae sp.) were observed.; recordedBy: Jui-Chen Hsieh; lifeStage: cocoon; occurrenceID: 4661FA09-66E7-58A0-81CE-E4E71D4EE33A; **Taxon:** taxonID: urn:lsid:zoobank.org:act:D8785F79-E874-4854-95D7-5C0A928914CA; scientificName: *Meteorusstellatus* Fujie, Shimizu & Maeto, 2021; acceptedNameUsage: *Meteorusstellatus* Fujie, Shimizu & Maeto, 2021; originalNameUsage: *Meteorusstellatus* Fujie, Shimizu & Maeto, 2021; kingdom: Animalia; phylum: Arthropoda; class: Insecta; order: Hymenoptera; family: Braconidae; genus: Meteorus; specificEpithet: *stellatus*; taxonRank: species; scientificNameAuthorship: Fujie, Shimizu & Maeto; vernacularName: ホシガタハラボソコマユバチ; nomenclaturalCode: ICZN; taxonomicStatus: accepted; **Location:** continent: Asia; islandGroup: Taiwan; island: Taiwan; country: Taiwan; countryCode: TW; county: Hsinchu; municipality: Emei township; locality: Tengping trail; **Identification:** identifiedBy: So Shimizu, Hsuan-Pu Chen, Kai-Ti Lin, Shunpei Fujie, Kaoru Maeto; dateIdentified: 2022; identificationReferences: Fujie et al. 2021; **Event:** eventDate: 30/12/2016; year: 2016; month: 12; day: 30; **Record Level:** type: StillImage; language: zh; basisOfRecord: HumanObservation; source: https://www.facebook.com/groups/369189783201470/permalink/1156066134513827/
- **Type status:** Other material. **Occurrence:** recordedBy: Hua-Ting Cheng; lifeStage: cocoon; occurrenceID: EE2F8D68-28EE-565D-AC39-5F426C81C03C; **Taxon:** taxonID: urn:lsid:zoobank.org:act:D8785F79-E874-4854-95D7-5C0A928914CA; scientificName: *Meteorusstellatus* Fujie, Shimizu & Maeto, 2021; acceptedNameUsage: *Meteorusstellatus* Fujie, Shimizu & Maeto, 2021; originalNameUsage: *Meteorusstellatus* Fujie, Shimizu & Maeto, 2021; kingdom: Animalia; phylum: Arthropoda; class: Insecta; order: Hymenoptera; family: Braconidae; genus: Meteorus; specificEpithet: *stellatus*; taxonRank: species; scientificNameAuthorship: Fujie, Shimizu & Maeto; vernacularName: ホシガタハラボソコマユバチ; nomenclaturalCode: ICZN; taxonomicStatus: accepted; **Location:** continent: Asia; islandGroup: Taiwan; island: Taiwan; country: Taiwan; countryCode: TW; **Identification:** identifiedBy: So Shimizu, Hsuan-Pu Chen, Kai-Ti Lin, Shunpei Fujie, Kaoru Maeto; dateIdentified: 2022; identificationReferences: Fujie et al. 2021; **Event:** eventDate: 11/07/2018; year: 2018; month: 7; day: 11; **Record Level:** type: StillImage; language: zh; basisOfRecord: HumanObservation; source: https://www.facebook.com/groups/369189783201470/permalink/1790064617780639/
- **Type status:** Other material. **Occurrence:** recordedBy: Ching-Chang Hsu; lifeStage: cocoon; occurrenceID: D76F0FAB-3758-55E2-8A64-21488DD15E4B; **Taxon:** taxonID: urn:lsid:zoobank.org:act:D8785F79-E874-4854-95D7-5C0A928914CA; scientificName: *Meteorusstellatus* Fujie, Shimizu & Maeto, 2021; acceptedNameUsage: *Meteorusstellatus* Fujie, Shimizu & Maeto, 2021; originalNameUsage: *Meteorusstellatus* Fujie, Shimizu & Maeto, 2021; kingdom: Animalia; phylum: Arthropoda; class: Insecta; order: Hymenoptera; family: Braconidae; genus: Meteorus; specificEpithet: *stellatus*; taxonRank: species; scientificNameAuthorship: Fujie, Shimizu & Maeto; vernacularName: ホシガタハラボソコマユバチ; nomenclaturalCode: ICZN; taxonomicStatus: accepted; **Location:** continent: Asia; islandGroup: Taiwan; island: Taiwan; country: Taiwan; countryCode: TW; stateProvince: New Taipei; municipality: Bali District; locality: Zhanshan trail, Mt. Guanyin; **Identification:** identifiedBy: So Shimizu, Hsuan-Pu Chen, Kai-Ti Lin, Shunpei Fujie, Kaoru Maeto; dateIdentified: 2022; identificationReferences: Fujie et al. 2021; **Event:** eventDate: 04/11/2018; year: 2018; month: 11; day: 4; **Record Level:** type: StillImage; language: zh; basisOfRecord: HumanObservation; source: https://www.facebook.com/groups/369189783201470/permalink/1969562113164221/
- **Type status:** Other material. **Occurrence:** occurrenceRemarks: Communal wasp cocoons; recordedBy: Shuling Yang; lifeStage: cocoon; occurrenceID: 88C217FD-1AF4-5389-8C9D-29B07087DAE7; **Taxon:** taxonID: urn:lsid:zoobank.org:act:D8785F79-E874-4854-95D7-5C0A928914CA; scientificName: *Meteorusstellatus* Fujie, Shimizu & Maeto, 2021; acceptedNameUsage: *Meteorusstellatus* Fujie, Shimizu & Maeto, 2021; originalNameUsage: *Meteorusstellatus* Fujie, Shimizu & Maeto, 2021; kingdom: Animalia; phylum: Arthropoda; class: Insecta; order: Hymenoptera; family: Braconidae; genus: Meteorus; specificEpithet: *stellatus*; taxonRank: species; scientificNameAuthorship: Fujie, Shimizu & Maeto; vernacularName: ホシガタハラボソコマユバチ; nomenclaturalCode: ICZN; taxonomicStatus: accepted; **Location:** continent: Asia; islandGroup: Taiwan; island: Taiwan; country: Taiwan; countryCode: TW; stateProvince: New Taipei; municipality: Yonghe; locality: Fuhe wetlands; **Identification:** identifiedBy: So Shimizu, Hsuan-Pu Chen, Kai-Ti Lin, Shunpei Fujie, Kaoru Maeto; dateIdentified: 2022; identificationReferences: Fujie et al. 2021; **Event:** eventDate: 12/11/2018; year: 2018; month: 11; day: 12; **Record Level:** type: StillImage; language: zh; basisOfRecord: HumanObservation
- **Type status:** Other material. **Occurrence:** occurrenceRemarks: Communal wasp cocoons; recordedBy: Shuling Yang; lifeStage: cocoon; occurrenceID: 54D85D60-F689-50E1-8065-66303B21B355; **Taxon:** taxonID: urn:lsid:zoobank.org:act:D8785F79-E874-4854-95D7-5C0A928914CA; scientificName: *Meteorusstellatus* Fujie, Shimizu & Maeto, 2021; acceptedNameUsage: *Meteorusstellatus* Fujie, Shimizu & Maeto, 2021; originalNameUsage: *Meteorusstellatus* Fujie, Shimizu & Maeto, 2021; kingdom: Animalia; phylum: Arthropoda; class: Insecta; order: Hymenoptera; family: Braconidae; genus: Meteorus; specificEpithet: *stellatus*; taxonRank: species; scientificNameAuthorship: Fujie, Shimizu & Maeto; vernacularName: ホシガタハラボソコマユバチ; nomenclaturalCode: ICZN; taxonomicStatus: accepted; **Location:** continent: Asia; islandGroup: Taiwan; island: Taiwan; country: Taiwan; countryCode: TW; stateProvince: Taipei; **Identification:** identifiedBy: So Shimizu, Hsuan-Pu Chen, Kai-Ti Lin, Shunpei Fujie, Kaoru Maeto; dateIdentified: 2022; identificationReferences: Fujie et al. 2021; **Event:** eventDate: 13/11/2018; year: 2018; month: 11; day: 13; **Record Level:** type: StillImage; language: zh; basisOfRecord: HumanObservation
- **Type status:** Other material. **Occurrence:** recordedBy: Shu-Ling Lin; lifeStage: cocoon; occurrenceID: A82F511C-9692-51E2-98E3-96B4222D0466; **Taxon:** taxonID: urn:lsid:zoobank.org:act:D8785F79-E874-4854-95D7-5C0A928914CA; scientificName: *Meteorusstellatus* Fujie, Shimizu & Maeto, 2021; acceptedNameUsage: *Meteorusstellatus* Fujie, Shimizu & Maeto, 2021; originalNameUsage: *Meteorusstellatus* Fujie, Shimizu & Maeto, 2021; kingdom: Animalia; phylum: Arthropoda; class: Insecta; order: Hymenoptera; family: Braconidae; genus: Meteorus; specificEpithet: *stellatus*; taxonRank: species; scientificNameAuthorship: Fujie, Shimizu & Maeto; vernacularName: ホシガタハラボソコマユバチ; nomenclaturalCode: ICZN; taxonomicStatus: accepted; **Location:** continent: Asia; islandGroup: Taiwan; island: Taiwan; country: Taiwan; countryCode: TW; county: Nantou; municipality: Lugu township; **Identification:** identifiedBy: So Shimizu, Hsuan-Pu Chen, Kai-Ti Lin, Shunpei Fujie, Kaoru Maeto; dateIdentified: 2022; identificationReferences: Fujie et al. 2021; **Event:** eventDate: 19/07/2018; year: 2018; month: 7; day: 19; **Record Level:** type: StillImage; language: zh; basisOfRecord: HumanObservation; source: https://www.facebook.com/groups/369189783201470/permalink/1804468623006905/
- **Type status:** Other material. **Occurrence:** occurrenceRemarks: Wasp cocoon was attacked by *Parapolybiavaria*.; recordedBy: Ke-Hsiung Tsai; lifeStage: cocoon; occurrenceID: A894D042-A6D6-5C78-8B5D-25731CCA2419; **Taxon:** taxonID: urn:lsid:zoobank.org:act:D8785F79-E874-4854-95D7-5C0A928914CA; scientificName: *Meteorusstellatus* Fujie, Shimizu & Maeto, 2021; acceptedNameUsage: *Meteorusstellatus* Fujie, Shimizu & Maeto, 2021; originalNameUsage: *Meteorusstellatus* Fujie, Shimizu & Maeto, 2021; kingdom: Animalia; phylum: Arthropoda; class: Insecta; order: Hymenoptera; family: Braconidae; genus: Meteorus; specificEpithet: *stellatus*; taxonRank: species; scientificNameAuthorship: Fujie, Shimizu & Maeto; vernacularName: ホシガタハラボソコマユバチ; nomenclaturalCode: ICZN; taxonomicStatus: accepted; **Location:** continent: Asia; islandGroup: Taiwan; island: Taiwan; country: Taiwan; countryCode: TW; county: Keelung; municipality: Xinyi District; locality: Gangziliao trail; **Identification:** identifiedBy: So Shimizu, Hsuan-Pu Chen, Kai-Ti Lin, Shunpei Fujie, Kaoru Maeto; dateIdentified: 2022; identificationReferences: Fujie et al. 2021; **Event:** eventDate: 01/04/2021; year: 2021; month: 4; day: 1; **Record Level:** type: StillImage; language: zh; basisOfRecord: HumanObservation; source: https://www.facebook.com/groups/393148477475231/permalink/4372047339585305/
- **Type status:** Other material. **Occurrence:** occurrenceRemarks: Wasp larvae and cocoons were reared from larva of *Macroglossumsitiene*.; recordedBy: Kai-Ti Lin; lifeStage: larva and cocoon; occurrenceID: 0778F8E3-5060-58DC-B5E2-1426FE87CCC0; **Taxon:** taxonID: urn:lsid:zoobank.org:act:D8785F79-E874-4854-95D7-5C0A928914CA; scientificName: *Meteorusstellatus* Fujie, Shimizu & Maeto, 2021; acceptedNameUsage: *Meteorusstellatus* Fujie, Shimizu & Maeto, 2021; originalNameUsage: *Meteorusstellatus* Fujie, Shimizu & Maeto, 2021; kingdom: Animalia; phylum: Arthropoda; class: Insecta; order: Hymenoptera; family: Braconidae; genus: Meteorus; specificEpithet: *stellatus*; taxonRank: species; scientificNameAuthorship: Fujie, Shimizu & Maeto; vernacularName: ホシガタハラボソコマユバチ; nomenclaturalCode: ICZN; taxonomicStatus: accepted; **Location:** continent: Asia; islandGroup: Taiwan; island: Taiwan; country: Taiwan; countryCode: TW; stateProvince: Taipei; municipality: Da'an District; locality: National Taiwan University; **Identification:** identifiedBy: So Shimizu, Hsuan-Pu Chen, Kai-Ti Lin, Shunpei Fujie, Kaoru Maeto; dateIdentified: 2022; identificationReferences: Fujie et al. 2021; **Event:** eventDate: 05/11/2021; year: 2021; month: 11; day: 5; **Record Level:** type: StillImage; language: zh; basisOfRecord: HumanObservation; source: https://www.facebook.com/groups/588350308851792/permalink/620323802321109/
- **Type status:** Other material. **Occurrence:** occurrenceRemarks: Wasp cocoon and tis hyperparasitoid wasp (Pteromalidae sp.) were observed.; recordedBy: Chun-Che Chien; lifeStage: cocoon; occurrenceID: AA46B1EC-4536-54A3-8FE6-EB7D9199E19A; **Taxon:** taxonID: urn:lsid:zoobank.org:act:D8785F79-E874-4854-95D7-5C0A928914CA; scientificName: *Meteorusstellatus* Fujie, Shimizu & Maeto, 2021; acceptedNameUsage: *Meteorusstellatus* Fujie, Shimizu & Maeto, 2021; originalNameUsage: *Meteorusstellatus* Fujie, Shimizu & Maeto, 2021; kingdom: Animalia; phylum: Arthropoda; class: Insecta; order: Hymenoptera; family: Braconidae; genus: Meteorus; specificEpithet: *stellatus*; taxonRank: species; scientificNameAuthorship: Fujie, Shimizu & Maeto; vernacularName: ホシガタハラボソコマユバチ; nomenclaturalCode: ICZN; taxonomicStatus: accepted; **Location:** continent: Asia; islandGroup: Taiwan; island: Taiwan; country: Taiwan; countryCode: TW; county: Pingtung; municipality: Sandimen township; locality: Mt. Dewun; **Identification:** identifiedBy: So Shimizu, Hsuan-Pu Chen, Kai-Ti Lin, Shunpei Fujie, Kaoru Maeto; dateIdentified: 2022; identificationReferences: Fujie et al. 2021; **Event:** eventDate: 09/11/2021; year: 2021; month: 11; day: 9; **Record Level:** type: StillImage; language: zh; basisOfRecord: HumanObservation; source: https://www.facebook.com/groups/393148477475231/permalink/4447480548708650/
- **Type status:** Other material. **Occurrence:** occurrenceRemarks: Wasp cocoon and its host larva (*Hippotioncelerio*) were observed.; recordedBy: Chean-Yueh Chang, Chun-Chung Su; lifeStage: cocoon; occurrenceID: 7616E757-8BE9-5BE5-A67F-8FDA0C8D8F33; **Taxon:** taxonID: urn:lsid:zoobank.org:act:D8785F79-E874-4854-95D7-5C0A928914CA; scientificName: *Meteorusstellatus* Fujie, Shimizu & Maeto, 2021; acceptedNameUsage: *Meteorusstellatus* Fujie, Shimizu & Maeto, 2021; originalNameUsage: *Meteorusstellatus* Fujie, Shimizu & Maeto, 2021; kingdom: Animalia; phylum: Arthropoda; class: Insecta; order: Hymenoptera; family: Braconidae; genus: Meteorus; specificEpithet: *stellatus*; taxonRank: species; scientificNameAuthorship: Fujie, Shimizu & Maeto; vernacularName: ホシガタハラボソコマユバチ; nomenclaturalCode: ICZN; taxonomicStatus: accepted; **Location:** continent: Asia; islandGroup: Taiwan; island: Taiwan; country: Taiwan; countryCode: TW; stateProvince: Taichung; municipality: Beitun District; **Identification:** identifiedBy: So Shimizu, Hsuan-Pu Chen, Kai-Ti Lin, Shunpei Fujie, Kaoru Maeto; dateIdentified: 2022; identificationReferences: Fujie et al. 2021; **Event:** eventDate: 29/11/2021; year: 2021; month: 11; day: 29; **Record Level:** type: StillImage; language: zh; basisOfRecord: HumanObservation; source: https://www.facebook.com/groups/369189783201470/permalink/4598695013584238/
- **Type status:** Other material. **Occurrence:** occurrenceRemarks: Wasp cocoon was attacked by *Parapolybiavaria*.; recordedBy: Su-Chuan Hung; lifeStage: cocoon; occurrenceID: 782B47EA-3846-5A14-A14B-6F09F3EC3BB1; **Taxon:** taxonID: urn:lsid:zoobank.org:act:D8785F79-E874-4854-95D7-5C0A928914CA; scientificName: *Meteorusstellatus* Fujie, Shimizu & Maeto, 2021; acceptedNameUsage: *Meteorusstellatus* Fujie, Shimizu & Maeto, 2021; originalNameUsage: *Meteorusstellatus* Fujie, Shimizu & Maeto, 2021; kingdom: Animalia; phylum: Arthropoda; class: Insecta; order: Hymenoptera; family: Braconidae; genus: Meteorus; specificEpithet: *stellatus*; taxonRank: species; scientificNameAuthorship: Fujie, Shimizu & Maeto; vernacularName: ホシガタハラボソコマユバチ; nomenclaturalCode: ICZN; taxonomicStatus: accepted; **Location:** continent: Asia; islandGroup: Taiwan; island: Taiwan; country: Taiwan; countryCode: TW; stateProvince: Taipei; municipality: Beitou District; locality: Erziping trail, Yangmingshan National Park; **Identification:** identifiedBy: So Shimizu, Hsuan-Pu Chen, Kai-Ti Lin, Shunpei Fujie, Kaoru Maeto; dateIdentified: 2022; identificationReferences: Fujie et al. 2021; **Event:** eventDate: 03/07/2022; year: 2022; month: 7; day: 3; **Record Level:** type: MovingImage; language: zh; basisOfRecord: HumanObservation; source: https://www.facebook.com/groups/588350308851792/permalink/775928626760625/
- **Type status:** Other material. **Occurrence:** occurrenceRemarks: Some cocoons were probably broken by their natural enemies.; recordedBy: Hwei-Shan Lai; lifeStage: cocoon; occurrenceID: 73B0277F-5317-56F9-9A23-CB6F73359975; **Taxon:** taxonID: urn:lsid:zoobank.org:act:D8785F79-E874-4854-95D7-5C0A928914CA; scientificName: *Meteorusstellatus* Fujie, Shimizu & Maeto, 2021; acceptedNameUsage: *Meteorusstellatus* Fujie, Shimizu & Maeto, 2021; originalNameUsage: *Meteorusstellatus* Fujie, Shimizu & Maeto, 2021; kingdom: Animalia; phylum: Arthropoda; class: Insecta; order: Hymenoptera; family: Braconidae; genus: Meteorus; specificEpithet: *stellatus*; taxonRank: species; scientificNameAuthorship: Fujie, Shimizu & Maeto; vernacularName: ホシガタハラボソコマユバチ; nomenclaturalCode: ICZN; taxonomicStatus: accepted; **Location:** continent: Asia; islandGroup: Taiwan; island: Taiwan; country: Taiwan; countryCode: TW; stateProvince: Taipei; municipality: Shihlin District; locality: Majiao historical trail; **Identification:** identifiedBy: So Shimizu, Hsuan-Pu Chen, Kai-Ti Lin, Shunpei Fujie, Kaoru Maeto; dateIdentified: 2022; identificationReferences: Fujie et al. 2021; **Event:** eventDate: 14/07/2022; year: 2022; month: 7; day: 14; **Record Level:** type: StillImage; language: zh; basisOfRecord: HumanObservation; source: https://www.facebook.com/groups/588350308851792/permalink/786089845744503/
- **Type status:** Other material. **Occurrence:** recordedBy: Sukenobu Konishi and Touta Takami; individualCount: 3; sex: 1 female and 2 males; lifeStage: cocoon and adult; occurrenceID: AAE66F6F-3D1A-5C9D-93F8-7F0BCB8C2507; **Taxon:** taxonID: urn:lsid:zoobank.org:act:D8785F79-E874-4854-95D7-5C0A928914CA; scientificName: *Meteorusstellatus* Fujie, Shimizu & Maeto, 2021; acceptedNameUsage: *Meteorusstellatus* Fujie, Shimizu & Maeto, 2021; originalNameUsage: *Meteorusstellatus* Fujie, Shimizu & Maeto, 2021; kingdom: Animalia; phylum: Arthropoda; class: Insecta; order: Hymenoptera; family: Braconidae; genus: Meteorus; specificEpithet: *stellatus*; taxonRank: species; scientificNameAuthorship: Fujie, Shimizu & Maeto; vernacularName: ホシガタハラボソコマユバチ; nomenclaturalCode: ICZN; taxonomicStatus: accepted; **Location:** continent: Asia; islandGroup: Japanese Archipelago; island: Yakushima; country: Japan; countryCode: JP; stateProvince: Kagoshima; county: Kumage; municipality: Yakushima Town, Nagakubo; **Identification:** identifiedBy: Shunpei Fujie, Kaoru Maeto, So Shimizu, Hsuan-Pu Chen, Kai-Ti Lin; dateIdentified: 2022; identificationReferences: Fujie et al. 2021; **Event:** eventDate: 29/10/2021; year: 2021; month: 10; day: 29; **Record Level:** type: StillImage; language: jp; basisOfRecord: HumanObservation
- **Type status:** Other material. **Occurrence:** occurrenceRemarks: Communal wasp cocoons; recordedBy: Ren-Jye Chen; lifeStage: cocoon; occurrenceID: 800E8465-E96A-56EC-A3FF-5F3130A91515; **Taxon:** taxonID: urn:lsid:zoobank.org:act:D8785F79-E874-4854-95D7-5C0A928914CA; scientificName: *Meteorusstellatus* Fujie, Shimizu & Maeto, 2021; acceptedNameUsage: *Meteorusstellatus* Fujie, Shimizu & Maeto, 2021; originalNameUsage: *Meteorusstellatus* Fujie, Shimizu & Maeto, 2021; kingdom: Animalia; phylum: Arthropoda; class: Insecta; order: Hymenoptera; family: Braconidae; genus: Meteorus; specificEpithet: *stellatus*; taxonRank: species; scientificNameAuthorship: Fujie, Shimizu & Maeto; vernacularName: ホシガタハラボソコマユバチ; nomenclaturalCode: ICZN; taxonomicStatus: accepted; **Location:** continent: Asia; islandGroup: Taiwan; island: Taiwan; country: Taiwan; countryCode: TW; county: Pingtung; municipality: Mudan Township; locality: Damei forest road; **Identification:** identifiedBy: Hsuan-Pu Chen; dateIdentified: 2022; identificationReferences: Fujie et al. 2021; **Event:** eventDate: 05/01/2003; year: 2003; month: 1; day: 5; **Record Level:** type: StillImage; language: zh; basisOfRecord: HumanObservation; source: https://www.facebook.com/notes/357500009028439/?hc_ref=ARSAoZn-XmWqo0I0NaS-w0z4G-qc7237NSyoHcTADmUqJEa-K7vyxTukjGUkrpvBGkc&fref=gs&dti=170749060193254&hc_location=group
- **Type status:** Other material. **Occurrence:** occurrenceRemarks: Wasp cocoon and its hyperparasitoid wasp (Pteromalidae sp.) were observed.; recordedBy: Ren-Jye Chen; lifeStage: cocoon; occurrenceID: 7614036B-7B94-5104-92B2-F419ED32310F; **Taxon:** taxonID: urn:lsid:zoobank.org:act:D8785F79-E874-4854-95D7-5C0A928914CA; scientificName: *Meteorusstellatus* Fujie, Shimizu & Maeto, 2021; acceptedNameUsage: *Meteorusstellatus* Fujie, Shimizu & Maeto, 2021; originalNameUsage: *Meteorusstellatus* Fujie, Shimizu & Maeto, 2021; kingdom: Animalia; phylum: Arthropoda; class: Insecta; order: Hymenoptera; family: Braconidae; genus: Meteorus; specificEpithet: *stellatus*; taxonRank: species; scientificNameAuthorship: Fujie, Shimizu & Maeto; vernacularName: ホシガタハラボソコマユバチ; nomenclaturalCode: ICZN; taxonomicStatus: accepted; **Location:** continent: Asia; islandGroup: Taiwan; island: Taiwan; country: Taiwan; countryCode: TW; stateProvince: Taoyuan; municipality: Daxi District; locality: Nanzigou trail; **Identification:** identifiedBy: Hsuan-Pu Chen; dateIdentified: 2022; identificationReferences: Fujie et al. 2021; **Event:** eventDate: 15/01/2018; year: 2018; month: 1; day: 15; **Record Level:** type: StillImage; language: zh; basisOfRecord: HumanObservation; source: https://www.facebook.com/notes/357500009028439/?hc_ref=ARSAoZn-XmWqo0I0NaS-w0z4G-qc7237NSyoHcTADmUqJEa-K7vyxTukjGUkrpvBGkc&fref=gs&dti=170749060193254&hc_location=group
- **Type status:** Other material. **Occurrence:** occurrenceRemarks: Wasp cocoon and its host larva (*Macroglossumpassalus*) were observed.; recordedBy: Ren-Jye Chen; lifeStage: cocoon; occurrenceID: 89F4A112-762D-5805-A881-E9C86A4224C0; **Taxon:** taxonID: urn:lsid:zoobank.org:act:D8785F79-E874-4854-95D7-5C0A928914CA; scientificName: *Meteorusstellatus* Fujie, Shimizu & Maeto, 2021; acceptedNameUsage: *Meteorusstellatus* Fujie, Shimizu & Maeto, 2021; originalNameUsage: *Meteorusstellatus* Fujie, Shimizu & Maeto, 2021; kingdom: Animalia; phylum: Arthropoda; class: Insecta; order: Hymenoptera; family: Braconidae; genus: Meteorus; specificEpithet: *stellatus*; taxonRank: species; scientificNameAuthorship: Fujie, Shimizu & Maeto; vernacularName: ホシガタハラボソコマユバチ; nomenclaturalCode: ICZN; taxonomicStatus: accepted; **Location:** continent: Asia; islandGroup: Taiwan; island: Taiwan; country: Taiwan; countryCode: TW; county: Pingtung; municipality: Shizi Township; locality: Shouka Forest Road; **Identification:** identifiedBy: Hsuan-Pu Chen; dateIdentified: 2022; identificationReferences: Fujie et al. 2021; **Event:** eventDate: 12/02/2023; year: 2023; month: 2; day: 12; **Record Level:** type: StillImage; language: zh; basisOfRecord: HumanObservation; source: https://www.facebook.com/photo?fbid=8976680589071832&set=a.395643457175631
- **Type status:** Other material. **Occurrence:** occurrenceRemarks: Wasp cocoon and its hyperparasitoid Darwin wasp (*Mesochorus* sp.) were observed.; recordedBy: Chun-Chun Deng; lifeStage: cocoon; occurrenceID: 6F7CAB51-8D6D-54B3-ABE2-9F9CF3152526; **Taxon:** taxonID: urn:lsid:zoobank.org:act:D8785F79-E874-4854-95D7-5C0A928914CA; scientificName: *Meteorusstellatus* Fujie, Shimizu & Maeto, 2021; acceptedNameUsage: *Meteorusstellatus* Fujie, Shimizu & Maeto, 2021; originalNameUsage: *Meteorusstellatus* Fujie, Shimizu & Maeto, 2021; kingdom: Animalia; phylum: Arthropoda; class: Insecta; order: Hymenoptera; family: Braconidae; genus: Meteorus; specificEpithet: *stellatus*; taxonRank: species; scientificNameAuthorship: Fujie, Shimizu & Maeto; vernacularName: ホシガタハラボソコマユバチ; nomenclaturalCode: ICZN; taxonomicStatus: accepted; **Location:** continent: Asia; islandGroup: Taiwan; island: Taiwan; country: Taiwan; countryCode: TW; stateProvince: Taipei; municipality: Da'an District; locality: Fujhoushan park; **Identification:** identifiedBy: So Shimizu, Hsuan-Pu Chen, Kai-Ti Lin, Shunpei Fujie, Kaoru Maeto; dateIdentified: 2022; identificationReferences: Fujie et al. 2021; **Event:** eventDate: 21/01/2022; year: 2022; month: 1; day: 21; **Record Level:** type: StillImage; language: zh; basisOfRecord: HumanObservation; source: https://www.facebook.com/groups/588350308851792/permalink/665203134499842/
- **Type status:** Other material. **Occurrence:** occurrenceRemarks: Adult wasps were emerged on 2023/1/2.; recordedBy: Alu Lu; lifeStage: cocoon; occurrenceID: 872DEDF7-1294-5C29-998B-66616302FB1E; **Taxon:** taxonID: urn:lsid:zoobank.org:act:D8785F79-E874-4854-95D7-5C0A928914CA; scientificName: *Meteorusstellatus* Fujie, Shimizu & Maeto, 2021; acceptedNameUsage: *Meteorusstellatus* Fujie, Shimizu & Maeto, 2021; originalNameUsage: *Meteorusstellatus* Fujie, Shimizu & Maeto, 2021; kingdom: Animalia; phylum: Arthropoda; class: Insecta; order: Hymenoptera; family: Braconidae; genus: Meteorus; specificEpithet: *stellatus*; taxonRank: species; scientificNameAuthorship: Fujie, Shimizu & Maeto; vernacularName: ホシガタハラボソコマユバチ; nomenclaturalCode: ICZN; taxonomicStatus: accepted; **Location:** continent: Asia; islandGroup: Taiwan; island: Taiwan; country: Taiwan; countryCode: TW; stateProvince: Taipei; municipality: Xindian District; **Identification:** identifiedBy: So Shimizu, Hsuan-Pu Chen, Kai-Ti Lin, Shunpei Fujie, Kaoru Maeto; dateIdentified: 2022; identificationReferences: Fujie et al. 2021; **Event:** eventDate: 23/12/2022; year: 2023; month: 12; day: 23; **Record Level:** type: StillImage; language: zh; basisOfRecord: HumanObservation; source: https://www.facebook.com/groups/588350308851792/permalink/891240715229415/
- **Type status:** Other material. **Occurrence:** recordedBy: Chun-Chun Deng; individualCount: 1; lifeStage: emerged cocoon; disposition: voucher NMNS; occurrenceID: 4E80B0A5-07FA-55BD-A9F2-F78F67466C52; **Taxon:** taxonID: urn:lsid:zoobank.org:act:D8785F79-E874-4854-95D7-5C0A928914CA; scientificName: *Meteorusstellatus* Fujie, Shimizu & Maeto, 2021; acceptedNameUsage: *Meteorusstellatus* Fujie, Shimizu & Maeto, 2021; originalNameUsage: *Meteorusstellatus* Fujie, Shimizu & Maeto, 2021; kingdom: Animalia; phylum: Arthropoda; class: Insecta; order: Hymenoptera; family: Braconidae; genus: Meteorus; specificEpithet: *stellatus*; taxonRank: species; scientificNameAuthorship: Fujie, Shimizu & Maeto; vernacularName: ホシガタハラボソコマユバチ; nomenclaturalCode: ICZN; taxonomicStatus: accepted; **Location:** continent: Asia; islandGroup: Taiwan; island: Taiwan; country: Taiwan; countryCode: TW; stateProvince: Taipei; municipality: Da'an District; locality: Fujhoushan park; **Identification:** identifiedBy: So Shimizu, Hsuan-Pu Chen, Kai-Ti Lin; dateIdentified: 2022; identificationReferences: Araujo et al. 2018; **Event:** eventDate: 21/01/2022; year: 2022; month: 1; day: 21; **Record Level:** type: PhysicalObject; basisOfRecord: PreservedSpecimen; source: https://www.facebook.com/groups/588350308851792/permalink/665203134499842/
- **Type status:** Other material. **Occurrence:** recordedBy: Ren-Jye Chen; individualCount: 1; lifeStage: emerged cocoon; disposition: voucher NMNS; occurrenceID: A529FE2D-D03D-5B10-8F8E-2EBAF3FD0076; **Taxon:** taxonID: urn:lsid:zoobank.org:act:D8785F79-E874-4854-95D7-5C0A928914CA; scientificName: *Meteorusstellatus* Fujie, Shimizu & Maeto, 2021; acceptedNameUsage: *Meteorusstellatus* Fujie, Shimizu & Maeto, 2021; originalNameUsage: *Meteorusstellatus* Fujie, Shimizu & Maeto, 2021; kingdom: Animalia; phylum: Arthropoda; class: Insecta; order: Hymenoptera; family: Braconidae; genus: Meteorus; specificEpithet: *stellatus*; taxonRank: species; scientificNameAuthorship: Fujie, Shimizu & Maeto; vernacularName: ホシガタハラボソコマユバチ; nomenclaturalCode: ICZN; taxonomicStatus: accepted; **Location:** continent: Asia; islandGroup: Taiwan; island: Taiwan; country: Taiwan; countryCode: TW; stateProvince: Taoyuan; municipality: Daxi District; locality: Nanzigou trail; **Identification:** identifiedBy: Hsuan-Pu Chen; dateIdentified: 2022; identificationReferences: Fujie et al. 2021; **Event:** eventDate: 15/01/2018; year: 2018; month: 1; day: 15; **Record Level:** type: PhysicalObject; basisOfRecord: PreservedSpecimen; source: https://www.facebook.com/notes/357500009028439/?hc_ref=ARSAoZn-XmWqo0I0NaS-w0z4G-qc7237NSyoHcTADmUqJEa-K7vyxTukjGUkrpvBGkc&fref=gs&dti=170749060193254&hc_location=group
- **Type status:** Other material. **Occurrence:** recordedBy: C. T. Yang (Chung-Tu Yang); individualCount: 45; sex: 34 females and 11 males; lifeStage: adult; disposition: voucher TARI; occurrenceID: EEE7F33C-D242-5C14-B95A-8324D2AE1CF2; **Taxon:** taxonID: urn:lsid:zoobank.org:act:D8785F79-E874-4854-95D7-5C0A928914CA; scientificName: *Meteorusstellatus* Fujie, Shimizu & Maeto, 2021; acceptedNameUsage: *Meteorusstellatus* Fujie, Shimizu & Maeto, 2021; originalNameUsage: *Meteorusstellatus* Fujie, Shimizu & Maeto, 2021; kingdom: Animalia; phylum: Arthropoda; class: Insecta; order: Hymenoptera; family: Braconidae; genus: Meteorus; specificEpithet: *stellatus*; taxonRank: species; scientificNameAuthorship: Fujie, Shimizu & Maeto; vernacularName: ホシガタハラボソコマユバチ; nomenclaturalCode: ICZN; taxonomicStatus: accepted; **Location:** continent: Asia; islandGroup: Taiwan; island: Taiwan; country: Taiwan; countryCode: TW; stateProvince: Taichung; **Identification:** identifiedBy: Kai-Ti Lin, Hsuan-Pu Chen; dateIdentified: 2022; identificationReferences: Fujie et al. 2021; **Event:** eventDate: 11/1990; year: 1990; month: 11; **Record Level:** type: PhysicalObject; basisOfRecord: PreservedSpecimen
- **Type status:** Other material. **Occurrence:** recordedBy: Jinhaku Sonan; individualCount: 37; sex: 26 females and 9 males; lifeStage: adult; disposition: voucher TARI; occurrenceID: 4DF0A521-C143-52D5-8E70-EEC35EDD4486; **Taxon:** taxonID: urn:lsid:zoobank.org:act:D8785F79-E874-4854-95D7-5C0A928914CA; scientificName: *Meteorusstellatus* Fujie, Shimizu & Maeto, 2021; acceptedNameUsage: *Meteorusstellatus* Fujie, Shimizu & Maeto, 2021; originalNameUsage: *Meteorusstellatus* Fujie, Shimizu & Maeto, 2021; kingdom: Animalia; phylum: Arthropoda; class: Insecta; order: Hymenoptera; family: Braconidae; genus: Meteorus; specificEpithet: *stellatus*; taxonRank: species; scientificNameAuthorship: Fujie, Shimizu & Maeto; vernacularName: ホシガタハラボソコマユバチ; nomenclaturalCode: ICZN; taxonomicStatus: accepted; **Location:** continent: Asia; islandGroup: Taiwan; island: Taiwan; country: Taiwan; countryCode: TW; locality: Sozan; locationRemarks: old locality name, equals to Yangmingshan, Beitou Dist., Taipei City, Taiwan; **Identification:** identifiedBy: Kai-Ti Lin, Hsuan-Pu Chen; dateIdentified: 2022; identificationReferences: Fujie et al. 2021; **Event:** eventDate: 22/06/1943; year: 1943; month: 6; day: 22; **Record Level:** type: PhysicalObject; basisOfRecord: PreservedSpecimen
- **Type status:** Other material. **Occurrence:** recordedBy: Jinhaku Sonan; individualCount: 1; lifeStage: emerged cocoon; disposition: voucher TARI; occurrenceID: A60C1D62-DC1D-54CF-AED9-775BE3D3B843; **Taxon:** taxonID: urn:lsid:zoobank.org:act:D8785F79-E874-4854-95D7-5C0A928914CA; scientificName: *Meteorusstellatus* Fujie, Shimizu & Maeto, 2021; acceptedNameUsage: *Meteorusstellatus* Fujie, Shimizu & Maeto, 2021; originalNameUsage: *Meteorusstellatus* Fujie, Shimizu & Maeto, 2021; kingdom: Animalia; phylum: Arthropoda; class: Insecta; order: Hymenoptera; family: Braconidae; genus: Meteorus; specificEpithet: *stellatus*; taxonRank: species; scientificNameAuthorship: Fujie, Shimizu & Maeto; vernacularName: ホシガタハラボソコマユバチ; nomenclaturalCode: ICZN; taxonomicStatus: accepted; **Location:** continent: Asia; islandGroup: Taiwan; island: Taiwan; country: Taiwan; countryCode: TW; locality: Sozan; locationRemarks: old locality name, equals to Yangmingshan, Beitou Dist., Taipei City, Taiwan; **Identification:** identifiedBy: Kai-Ti Lin, Hsuan-Pu Chen; dateIdentified: 2022; identificationReferences: Fujie et al. 2021; **Event:** eventDate: 22/06/1943; year: 1943; month: 6; day: 22; **Record Level:** type: PhysicalObject; basisOfRecord: PreservedSpecimen
- **Type status:** Other material. **Occurrence:** recordedBy: Kai-Ti Lin; lifeStage: adult; disposition: voucher TARI; occurrenceID: 30B047C2-A6AE-5BA9-B515-14E0101E644D; **Taxon:** taxonID: urn:lsid:zoobank.org:act:D8785F79-E874-4854-95D7-5C0A928914CA; scientificName: *Meteorusstellatus* Fujie, Shimizu & Maeto, 2021; acceptedNameUsage: *Meteorusstellatus* Fujie, Shimizu & Maeto, 2021; originalNameUsage: *Meteorusstellatus* Fujie, Shimizu & Maeto, 2021; kingdom: Animalia; phylum: Arthropoda; class: Insecta; order: Hymenoptera; family: Braconidae; genus: Meteorus; specificEpithet: *stellatus*; taxonRank: species; scientificNameAuthorship: Fujie, Shimizu & Maeto; vernacularName: ホシガタハラボソコマユバチ; nomenclaturalCode: ICZN; taxonomicStatus: accepted; **Location:** continent: Asia; islandGroup: Taiwan; island: Taiwan; country: Taiwan; countryCode: TW; stateProvince: Taipei; municipality: Da'an District; locality: National Taiwan University; **Identification:** identifiedBy: So Shimizu, Hsuan-Pu Chen, Kai-Ti Lin, Shunpei Fujie, Kaoru Maeto; dateIdentified: 2022; identificationReferences: Fujie et al. 2021; **Event:** eventDate: 05/11/2021; year: 2021; month: 11; day: 5; **Record Level:** type: PhysicalObject; basisOfRecord: PreservedSpecimen; source: https://www.facebook.com/groups/588350308851792/permalink/620323802321109/
- **Type status:** Other material. **Occurrence:** recordedBy: Sukenobu Konishi and Touta Takami; individualCount: 3; sex: 1 female and 2 males; lifeStage: cocoon and adult; disposition: voucher KPM; occurrenceID: 79F648BD-5C25-5AFD-A159-8CDDE266885F; **Taxon:** taxonID: urn:lsid:zoobank.org:act:D8785F79-E874-4854-95D7-5C0A928914CA; scientificName: *Meteorusstellatus* Fujie, Shimizu & Maeto, 2021; acceptedNameUsage: *Meteorusstellatus* Fujie, Shimizu & Maeto, 2021; originalNameUsage: *Meteorusstellatus* Fujie, Shimizu & Maeto, 2021; kingdom: Animalia; phylum: Arthropoda; class: Insecta; order: Hymenoptera; family: Braconidae; genus: Meteorus; specificEpithet: *stellatus*; taxonRank: species; scientificNameAuthorship: Fujie, Shimizu & Maeto; vernacularName: ホシガタハラボソコマユバチ; nomenclaturalCode: ICZN; taxonomicStatus: accepted; **Location:** continent: Asia; islandGroup: Japanese Archipelago; island: Yakushima; country: Japan; countryCode: JP; stateProvince: Kagoshima; county: Kumage; municipality: Yakushima Town, Nagakubo; **Identification:** identifiedBy: Shunpei Fujie, Kaoru Maeto, So Shimizu, Hsuan-Pu Chen, Kai-Ti Lin; dateIdentified: 2022; identificationReferences: Fujie et al. 2021; **Event:** eventDate: 29/10/2021; year: 2021; month: 10; day: 29; **Record Level:** type: PhysicalObject; basisOfRecord: PreservedSpecimen

#### Diagnosis

See the "Taxon treatment" section of Fujie et al. (2021).

#### Distribution

**Figure 1.**
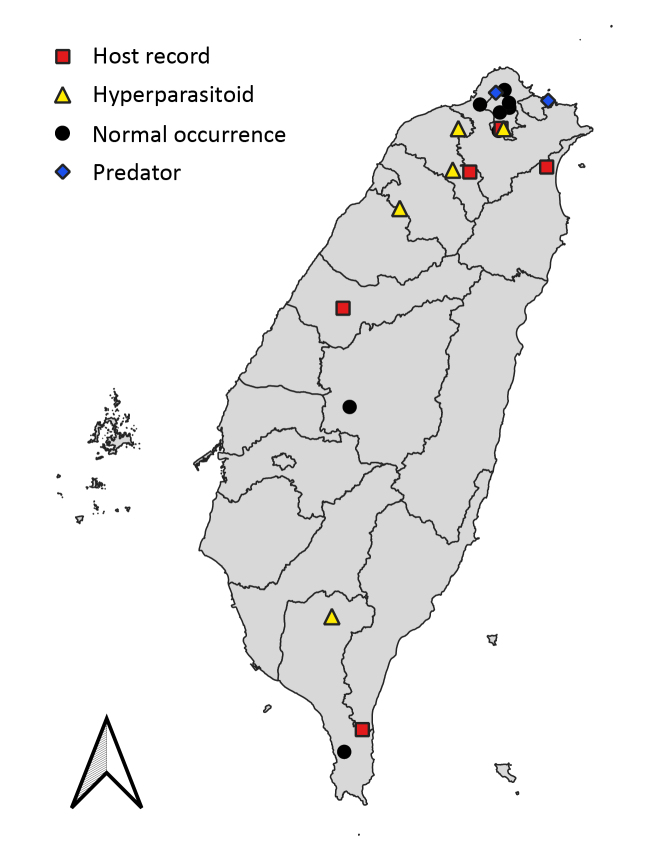
Distribution map of digital occurrence data of *Meteorusstellatus* Fujie, Shimizu & Maeto, 2021 (Hymenoptera, Braconidae, Euphorinae) from Taiwan. Red square: occurrence data with host record, yellow triangle: occurrence data for those attacked by hyperparasitoids, black circle: normal occurrence data, blue rhombus: occurrence data for those attacked by predators.

Eastern Palaearctic (Japan) (present paper) and Oriental Regions (Japan and Taiwan) (Fujie et al. 2021; present paper, Fig. 1).

#### Occurrence data of *M.stellatus*

**Figure 2.**
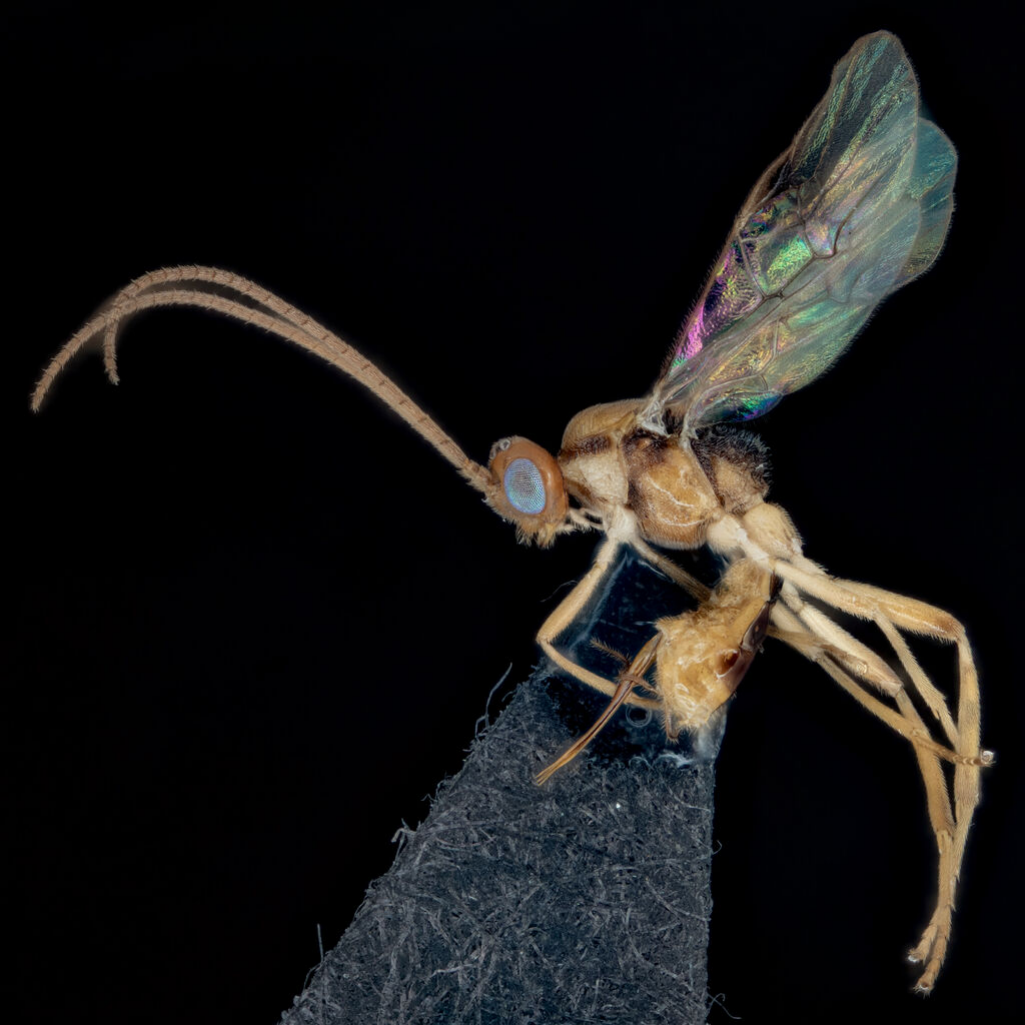
A female *Meteorusstellatus* Fujie, Shimizu & Maeto, 2021 (Hymenoptera, Braconidae, Euphorinae) reared from *Macroglossumsitiene* (Walker, 1856) (Lepidoptera, Sphingidae) by Kai-Ti Lin in Taiwan (photographed by So Shimizu).

**Figure 3.**
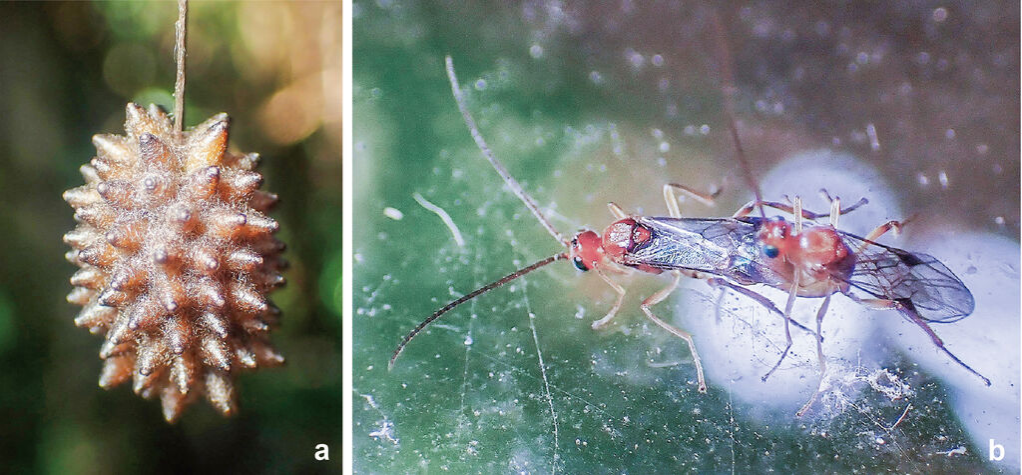
Source photographs of digital occurrence data of *Meteorusstellatus* Fujie, Shimizu & Maeto, 2021 (Hymenoptera, Braconidae, Euphorinae) from Taiwan observed by Alu Lu on 23.XII.2022: **a.** suspended communal cocoon; **b.** mating behaviour.

**Figure 4.**
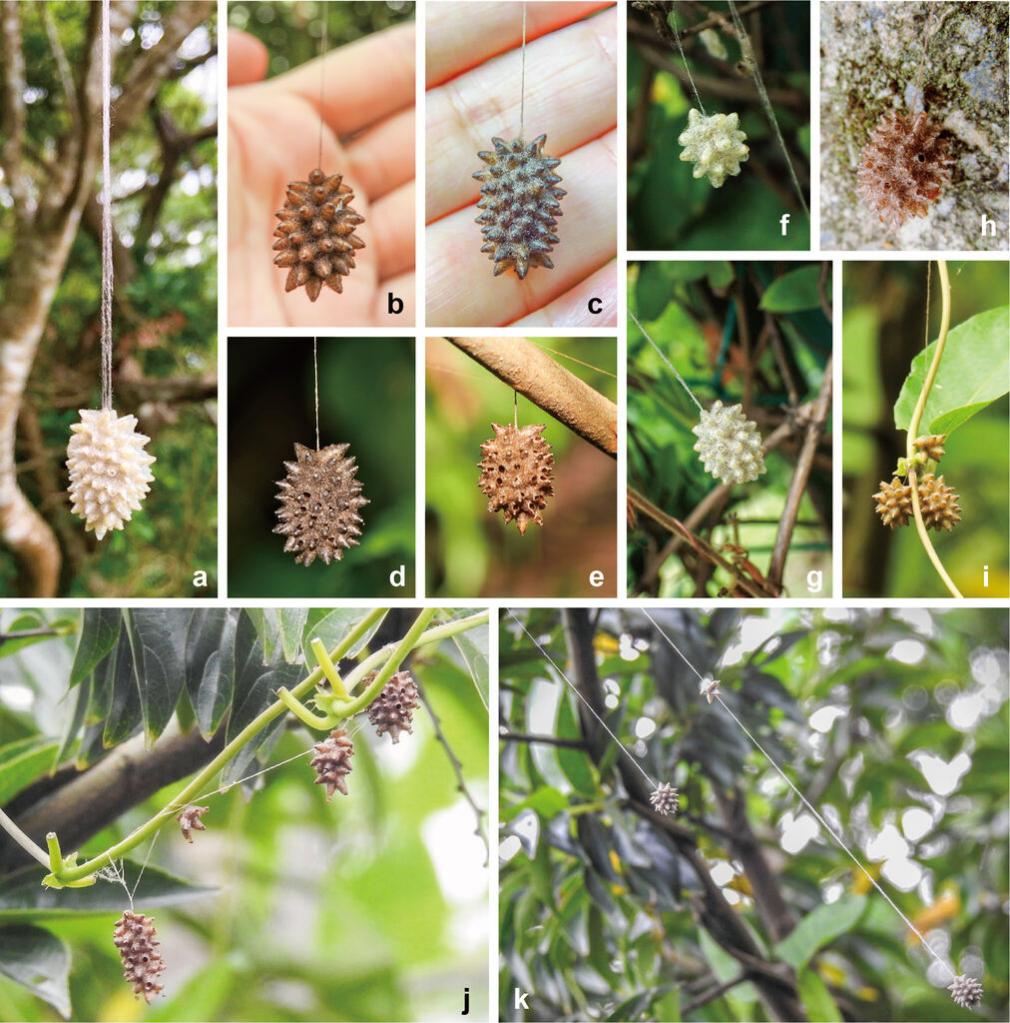
Source photographs of digital occurrence data of *Meteorusstellatus* Fujie, Shimizu & Maeto, 2021 (Hymenoptera, Braconidae, Euphorinae) from Taiwan, based on suspended communal cocoons: **a.** photographed by Ching-Chang Hsu on 4.XI.2018 in Zhanshan trail, Mt. Guanyin, Bali Dist., New Taipei City; **b.** photographed by Hua-Ting Cheng on 11.VII.2018 in Taiwan; **c.** photographed by Shu-Ling Lin on 19.VII.2018 in Lugu township, Nantou County; **d.** photographed by Hwei-Shan Lai on 14.VII.2022 in Majiao historical trail, Shihlin Dist., Taipei City; **e.** photographed by Shu-Ping Yang on 24.VIII.2013 in Mt. Dalunwei, Shihlin Dist., Taipei City; **f-g.** photographed by Shuling Yang on 13.XI.2018 in Taipei City; **h.** photographed by Tieh Hu (胡蝶) on 7.XII.2013 in Yangmingshan National Park, Beitou Dist., Taipei City; **i-k.** photographed by Shuling Yang on 12.XI.2018 in Fuhe wetlands, Yonghe Dist., New Taipei City.

**Figure 5.**
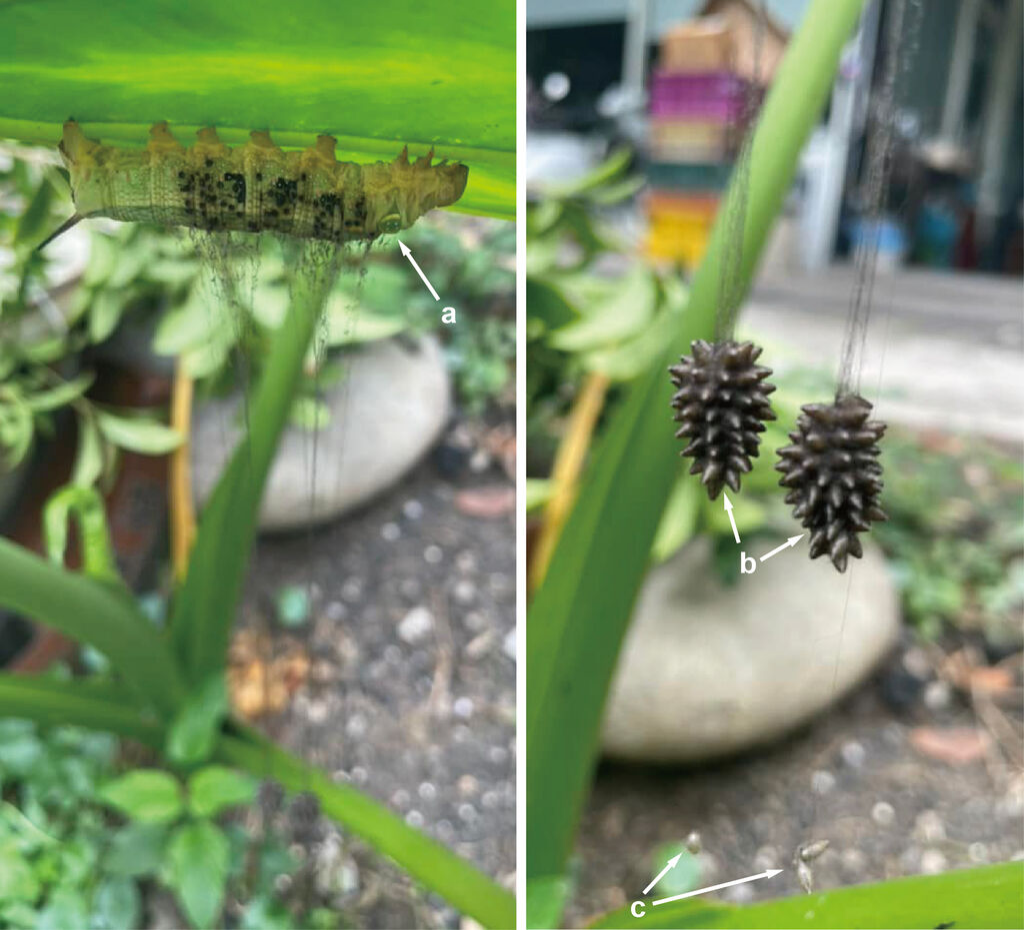
Source photographs of digital occurrence data of *Meteorusstellatus* Fujie, Shimizu & Maeto, 2021 (Hymenoptera, Braconidae, Euphorinae) and its host moth of *Hippotioncelerio* (Linnaeus, 1758) (Lepidoptera, Sphingidae) from Taiwan observed by Chean-Yueh Chang & Chun-Chung Su on 29.XI.2021: **a.** *H.celerio* parasitised by *M.stellatus*; **b.** large communal cocoons of *M.stellatus*; **c.** small cocoons of *M.stellatus*.

**Figure 6.**
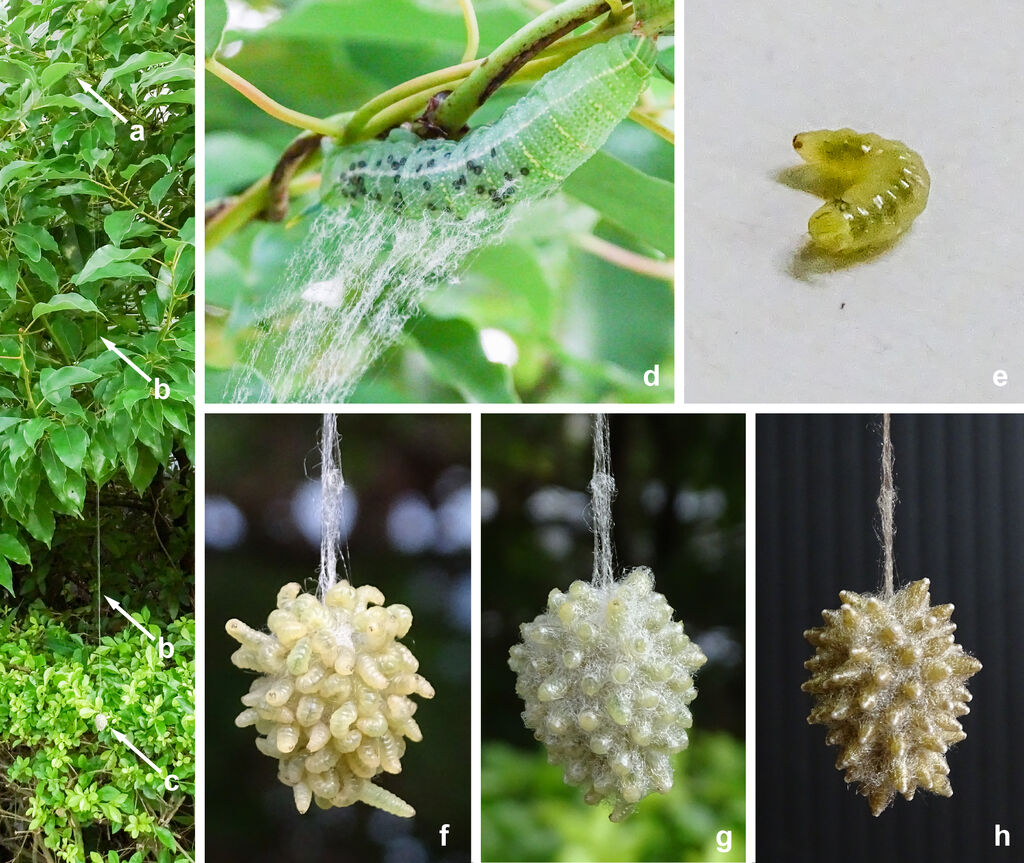
Source photographs of digital occurrence data of *Meteorusstellatus* Fujie, Shimizu & Maeto, 2021 (Hymenoptera, Braconidae, Euphorinae) and its host moth of *Macroglossumsitiene* (Walker, 1856) (Lepidoptera, Sphingidae) from Taiwan observed by Kai-Ti Lin on 5.XI.2021: **a.** upper end of a long cable of communal cocoon of *M.stellatus*; **b.** long cable of communal cocoon of *M.stellatus*; **c.** suspended communal cocoon of *M.stellatus* by a long cable; **d.** host larva of *Ma.sitiene*; **e.** larva of *M.stellatus*; **f.** early stage of communal cocoon construction behaviour of *M.stellatus*; **g.** middle stage of communal cocoon construction behaviour of *M.stellatus*; **h.** final stage of communal cocoon construction behaviour of *M.stellatus*.

**Figure 7.**
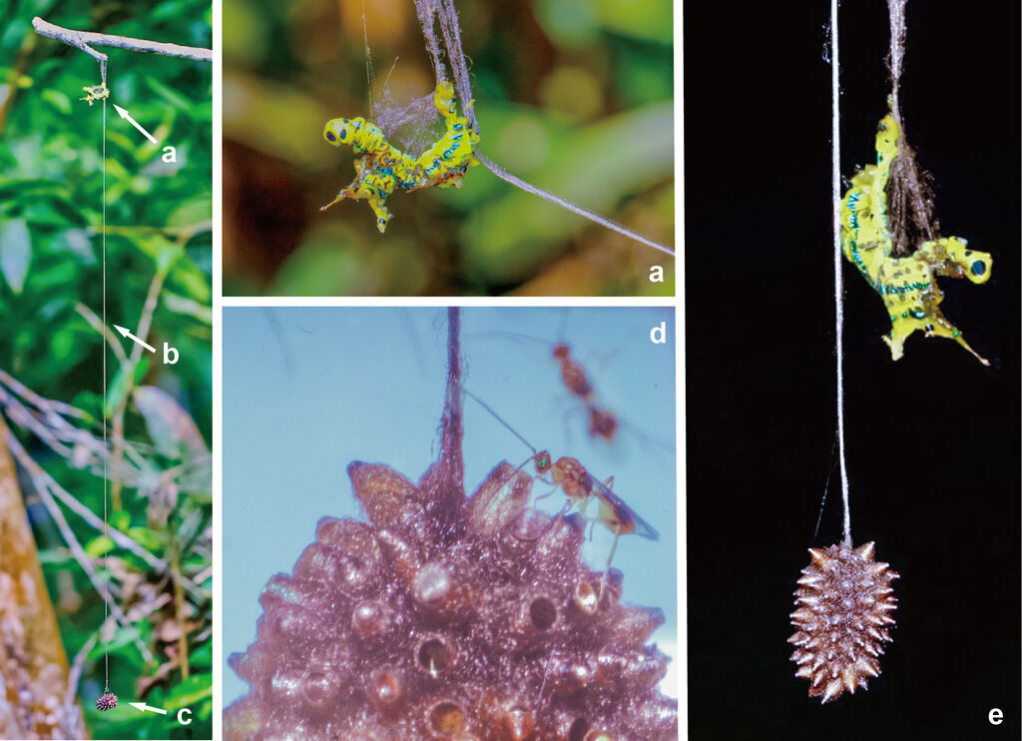
Source photographs of digital occurrence data of *Meteorusstellatus* Fujie, Shimizu & Maeto, 2021 (Hymenoptera, Braconidae, Euphorinae) and its host moth of *Macroglossumpassalus* (Drury, 1773) (Lepidoptera, Sphingidae) from Taiwan observed by Ren-Jye Chen on 12.II.2023: **a.** upper end of a long cable of a communal cocoon of *M.stellatus* with host larva of *Ma.passalus*; **b.** long cable of *M.stellatus*; **c.** lower end of a long cable of a communal cocoon of *M.stellatus*; **d.** communal cocoon and emerged adult wasps of *M.stellatus*; **e.** communal cocoon of *M.stellatus* with host larva of *Ma.passalus*.

**Figure 8.**
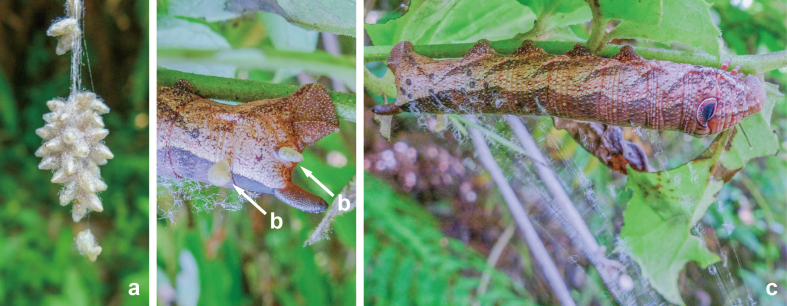
Source photographs of digital occurrence data of *Meteorusstellatus* Fujie, Shimizu & Maeto, 2021 (Hymenoptera, Braconidae, Euphorinae) and its host moth of *Cechetraminor* (Butler, 1875) (Lepidoptera, Sphingidae) from Taiwan observed by Mei-Ling Lo on 14.VII.2012: **a.** communal cocoon of *M.stellatus*; **b.** emerging larvae of *M.stellatus* from thier host body; **c.** host moth larva of *M.stellatus*.

**Figure 9.**
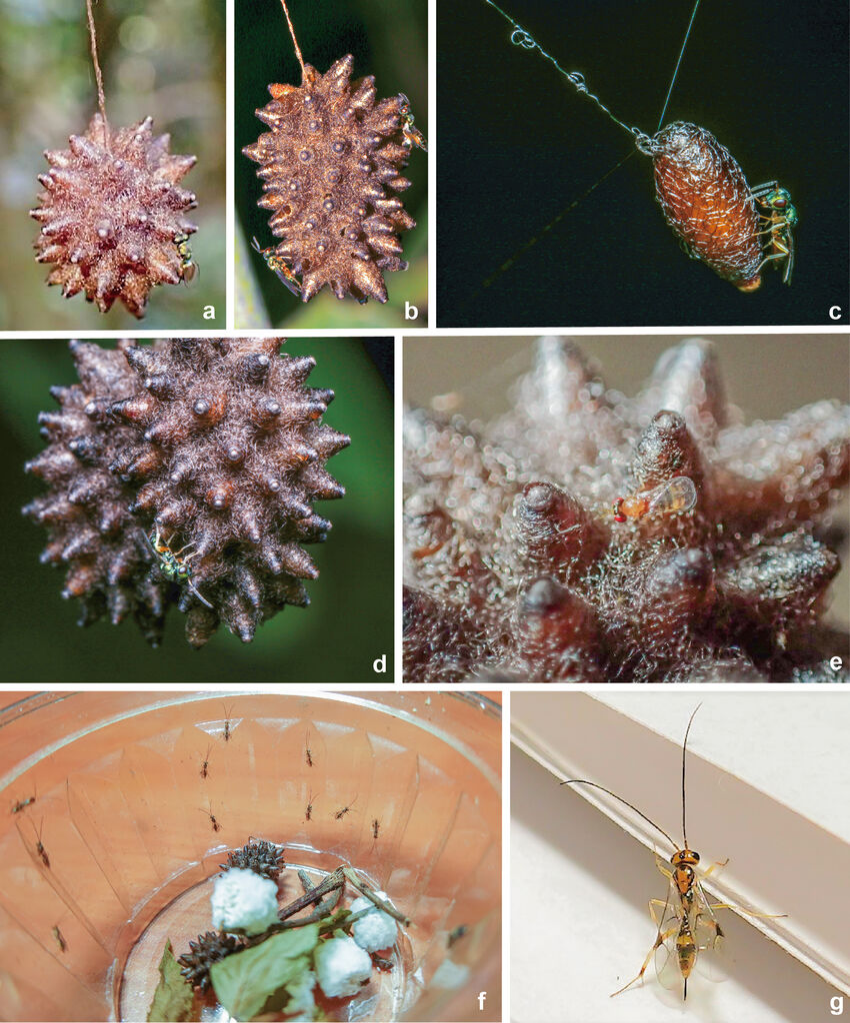
Source photographs of digital occurrence data of hyperparasitoid wasps of *Meteorusstellatus* Fujie, Shimizu & Maeto, 2021 (Hymenoptera, Braconidae, Euphorinae) from Taiwan observed by Taiwanese citizen scientists: **a.** communal cocoon of *M.stellatus* and adult hyperparasitoid wasp of Pteromalidae (photographed by Jui-Chen Hsieh on 30.II.2016); **b.** communal cocoon of *M.stellatus* and adult hyperparasitoid wasp of Pteromalidae (photographed by Ren-Jye Chen on 15.I.2018); **c-d.** communal cocoon of *M.stellatus* and adult hyperparasitoid wasp of Pteromalidae (photographed by Chun-Che Chien on 9.XI.2021); **e.** communal cocoon of *M.stellatus* and adult hyperparasitoid wasp of Trichogrammatidae (photographed by Mei-Ling Lo on 23.XII.2015); **f-g.** communal cocoon of *M.stellatus* and emerged adult hyperparasitoid wasps of the Darwin wasp genus *Mesochorus* sp. (photographed by Chun-Chun Deng on 21.I.2022).

**Figure 10.**
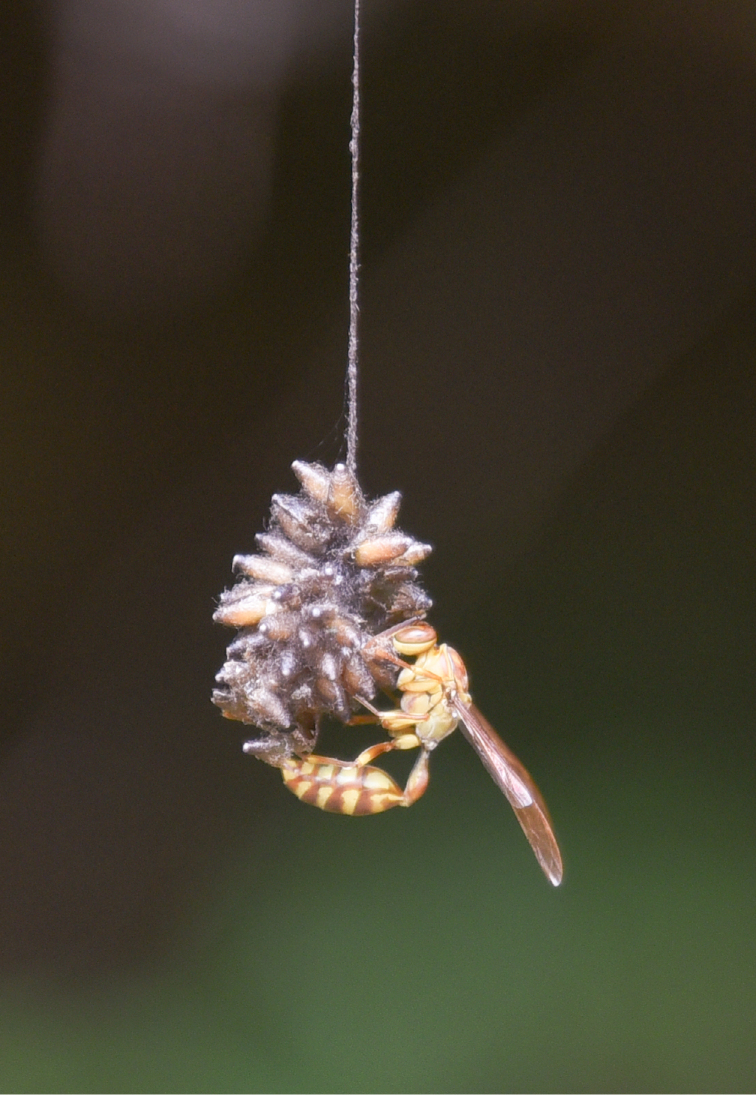
Source photograph of digital occurrence data of predator of *Meteorusstellatus* Fujie, Shimizu & Maeto, 2021 (Hymenoptera, Braconidae, Euphorinae) from Taiwan observed by Ke-Hsiung Tsai. A female paper wasp worker of *Parapolybiavaria* (Fabricius, 1787) (Hymenoptera, Vespidae) attacking a suspended communal cocoon of *M.stellatus*.

**Figure 11.**
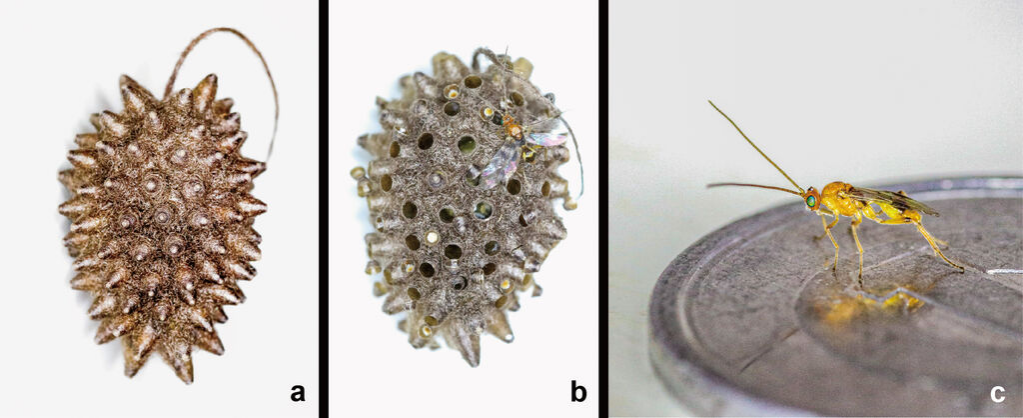
Source photographs of digital occurrence data of *Meteorusstellatus* Fujie, Shimizu & Maeto, 2021 (Hymenoptera, Braconidae, Euphorinae) from Yakushima Is., Japan (photographed by Sukenobu Konishi and Touta Takami in 2021): **a.** communal cocoon before emerging adult wasps; **b.** communal cocoon after emerging adult wasps; **c.** emerged female adult wasp on a one-yen coin.

A total of 25 digital occurrence data of *M.stellatus* were compiled. Twenty-two of them were permitted by the posters for reuse of their data in the present paper (Table 3), while three were not. Amongst the permitted data, 21 were obtained from the Taiwanese Facebook posts, representing the first record of *M.stellatus* from Taiwan (Table 3; Figs 1, 2, 3, 4, 5, 6, 7, 8, 9, 10). One digital occurrence record was recognised from Yakushima Is. Japan, via the Japanese website, representing the first record of *M.stellatus* from the Eastern Palaearctic Region (Table 3; Fig. 11). The suspended communal cocoons were observed in all compiled data (Table 3), but larval and adult stages were not. In addition, associated insects with *M.stellatus* were recognised in 11 of the 22 permitted digital occurrence data (50% of all data) (see Table 3 and the below "Insects associated with *M.stellatus*" section).

**Figure 12.**
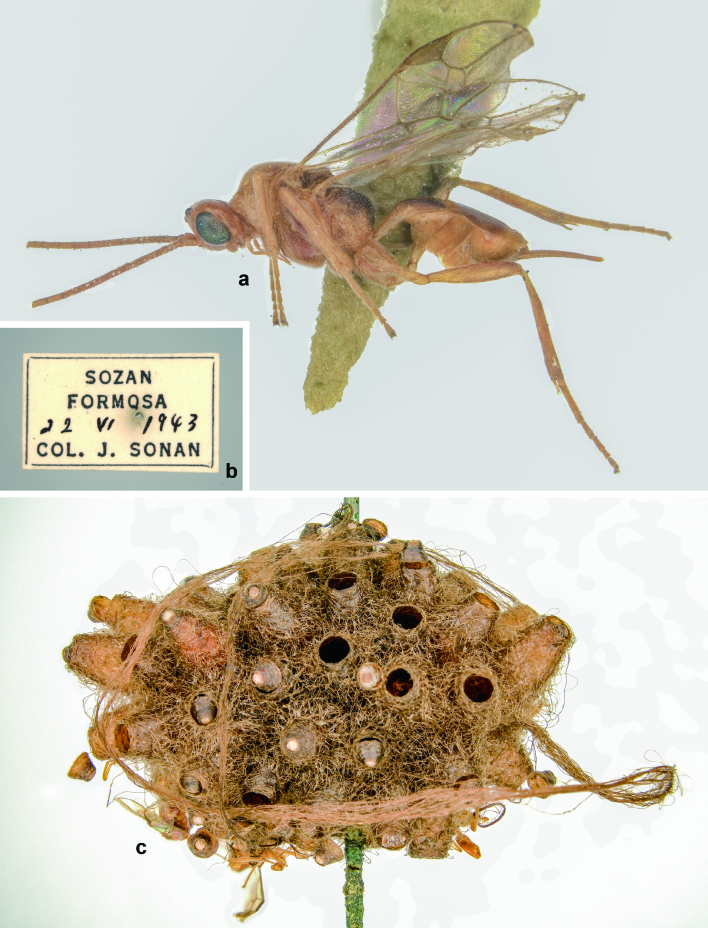
*Meteorusstellatus* Fujie, Shimizu & Maeto, 2021 (Hymenoptera, Braconidae, Euphorinae) from Taiwan found in the J. Sonan collection at TARI (photographed by Hsuan-Pu Chen): **a.** female adult; **b.** data label; **c.** communal cocoon.

Seven physical occurrence data were also listed in the present paper (Table 4; Figs 2, 12). Four of them were based on voucher-preserved specimens for digital occurrence data, while the remaining three were recognised through investigation of a Hymenoptera collection preserved at TARI by HPC and KTL.

#### Insects associated with *M.stellatus*

A total of 12 insect taxa were recognised as being associated with *M.stellatus* (Table 5) (including the data from Fujie et al. (2021)).

##### Hosts

A total of five host species from three hawk moth genera (Lepidoptera, Sphingidae: *Cechetra*, *Hippotion* and *Macroglossum*) were listed in Table 5. Three of which (*C.minor*, *H.celerio* and *Ma.sitiene*) were recorded for the first time as hosts. The majority of moth species currently recognised as hosts belong to the genus *Macroglossum*. On the other hand, two host moth species belong to the genera *Cechetra* and *Hippotion*, representing the first genus-level host records for *M.stellatus*. As hosts of *M.stellatus*, three (*C.minor*, *H.celerio* and *Ma.sitiene*) were known only from Taiwan (present paper), one (*Ma.passalus*) was from both Taiwan (present paper) and Japan (Fujie et al. 2021) and one (*Ma.pyrrhosticta*) was only from Japan (Fujie et al. 2021).

##### Hyperparasitoids

A total of six hyperparasitoid wasps, including three new data from Taiwan, were listed in Table 5. However, two of the new data could unfortunately not be identified as generic- and species-levels and were excluded from the "Taxon treatments" section in the present paper, while their higher classification (indeterminate species of Pteromalidae and Trichogrammatidae families) was mentioned in the "occurrenceRemarks" of *M.stellatus* and Table 5. The remaining new hyperparasitoid wasp data were identified as the Darwin wasp genus *Mesochorus* (Ichneumonidae, Mesochorinae), but the specific name could not be identified.

##### Predators

Only one paper wasp species, *Parapolybiavaria* (Fabricius, 1787) (Hymenoptera, Vespidae), was recognised as a predator (Table 5; Fig. 10). It was recognised from two sources (Table 3). The cocoon of *M.stellatus* was intensively attacked by either a single worker (Fig. 10) or many workers (as seen in the video at https://www.youtube.com/watch?v=AYzqgeJwxOo). The communal cocoon, suspended by a long cable, was moderately to strongly swaying and spinning due to natural winds and the flapping of the paper wasps' wings. First, the paper wasps were hovering in the air and trying to figure out the optimal timing for landing on the cocoon. Subsequently, they landed on the cocoon if it was relatively stable, but gave up landing on it if it was unstable.

### Cechetra minor

(Butler, 1875)

CD5882E6-676B-5CB4-ACD4-BEFBFC999542

https://www.gbif.org/species/165296887

#### Materials

- **Type status:** Other material. **Occurrence:** occurrenceRemarks: Moth larva and its parasitoid (*Meteorusstellatus*) were observed.; recordedBy: Mei-Ling Lo; lifeStage: larva; occurrenceID: BC78F277-9BE8-5FEF-8EC4-A56BD39AF4A4; **Taxon:** scientificName: *Cechetraminor* (Butler, 1875); acceptedNameUsage: *Cechetraminor* (Butler, 1875); originalNameUsage: *Chaerocampaminor* Butler, 1875; kingdom: Animalia; phylum: Arthropoda; class: Insecta; order: Lepidoptera; family: Sphingidae; genus: Cechetra; specificEpithet: minor; taxonRank: species; scientificNameAuthorship: Butler; vernacularName: 背線天蛾; nomenclaturalCode: ICZN; taxonomicStatus: accepted; **Location:** continent: Asia; islandGroup: Taiwan; island: Taiwan; country: Taiwan; countryCode: TW; stateProvince: Taoyuan; municipality: Fuxing; locality: Xiaoyun Elementary School, Xiayun; **Identification:** identifiedBy: Mei-Ling Lo; dateIdentified: 2022; identificationReferences: Chen 1994, Wang 1995; **Event:** eventDate: 14/07/2012; year: 2012; month: 7; day: 14; **Record Level:** type: StillImage; language: zh; basisOfRecord: HumanObservation; source: https://www.facebook.com/groups/588350308851792/permalink/627584148261741/

#### Taxon discussion

The larva of this species was identified, based on Chen (1994) and Wang (1995).

#### Notes

Newly recognised as a host of *M.stellatus*.

### Hippotion celerio

(Linnaeus, 1758)

065159AC-1CD7-578D-B336-CE8F4FDBC65B

https://www.gbif.org/species/1862293

#### Materials

- **Type status:** Other material. **Occurrence:** occurrenceRemarks: Moth larva and its parasitoid (*Meteorusstellatus*) were observed.; recordedBy: Chean-Yueh Chang, Chun-Chung Su; lifeStage: larva; occurrenceID: 352FCD94-8192-59B6-AD01-D53C7039B561; **Taxon:** scientificName: *Hippotioncelerio* (Linnaeus, 1758); acceptedNameUsage: *Hippotioncelerio* (Linnaeus, 1758); originalNameUsage: *Sphinxcelerio* Linnaeus, 1758; kingdom: Animalia; phylum: Arthropoda; class: Insecta; order: Lepidoptera; family: Sphingidae; genus: Hippotion; specificEpithet: celerio; taxonRank: species; scientificNameAuthorship: Linnaeus; vernacularName: シタベニセスジスズメ, 銀條斜線天蛾; nomenclaturalCode: ICZN; taxonomicStatus: accepted; **Location:** continent: Asia; islandGroup: Taiwan; island: Taiwan; country: Taiwan; countryCode: TW; stateProvince: Taichung; municipality: Beitun; **Identification:** identifiedBy: Shipher Wu; dateIdentified: 2022; identificationReferences: Chen 1994, Wang 1995; **Event:** eventDate: 29/11/2021; year: 2021; month: 11; day: 29; **Record Level:** type: StillImage; language: zh; basisOfRecord: HumanObservation; source: https://www.facebook.com/groups/369189783201470/permalink/4598695013584238/

#### Taxon discussion

The larva of this species was identified, based on Chen (1994) and Wang (1995).

#### Notes

Newly recognised as a host of *M.stellatus*.

### Macroglossum passalus

(Drury, 1773)

D36DBB5C-17D9-52A3-83AE-27CFDA901697

https://eol.org/pages/405563

https://www.gbif.org/species/5124305

#### Materials

- **Type status:** Other material. **Occurrence:** occurrenceRemarks: Moth larva and its parasitoid (*Meteorusstellatus*) were observed.; recordedBy: Ren-Jye Chen; lifeStage: larva; occurrenceID: CF350B71-1D14-5101-8959-249FBB9B87A1; **Taxon:** scientificName: *Macroglossumpassalus* (Drury, 1773); acceptedNameUsage: *Macroglossumpassalus* (Drury, 1774); originalNameUsage: *Sphinxpassalus* Drury, 1773; kingdom: Animalia; phylum: Arthropoda; class: Insecta; order: Lepidoptera; family: Sphingidae; genus: Macroglossum; specificEpithet: passalus; taxonRank: species; scientificNameAuthorship: Drury; vernacularName: 虎皮楠長喙天蛾; nomenclaturalCode: ICZN; taxonomicStatus: accepted; **Location:** continent: Asia; islandGroup: Taiwan; island: Taiwan; country: Taiwan; countryCode: TW; county: Pingtung; municipality: Shizi Township; locality: Shouka Forest Road; **Identification:** identifiedBy: Ren-Jye Chen; dateIdentified: 2022; identificationReferences: Chen 1994, Wang 1995; **Event:** eventDate: 12/02/2023; year: 2023; month: 2; day: 12; **Record Level:** type: StillImage; language: zh; basisOfRecord: HumanObservation; source: https://www.facebook.com/photo?fbid=8976680589071832&set=a.395643457175631

#### Taxon discussion

The larva of this species was identified, based on Chen (1994) and Wang (1995).

#### Notes

Known as a host of *M.stellatus* from Japan (Fujie et al. 2021) and Taiwan (present paper).

### Macroglossum sitiene

(Walker, 1856)

06E5D5D5-1669-5BB2-AD11-7349FAFA4862

https://www.gbif.org/species/5124374

#### Materials

- **Type status:** Other material. **Occurrence:** occurrenceRemarks: Moth larva and its parasitoid (*Meteorusstellatus*) were observed.; recordedBy: Kai-Ti Lin; lifeStage: larva; occurrenceID: 59D258E4-19EC-53A9-B1B9-434F00614DDE; **Taxon:** scientificName: *Macroglossumsitiene* (Walker, 1856); acceptedNameUsage: *Macroglossumsitiene* (Walker, 1856); originalNameUsage: *Macroglossasitiene* Walker, 1856; kingdom: Animalia; phylum: Arthropoda; class: Insecta; order: Lepidoptera; family: Sphingidae; genus: Macroglossum; specificEpithet: *pyrrhosticta*; taxonRank: species; scientificNameAuthorship: Walker; vernacularName: クロオビホウジャク, 膝帶長喙天蛾; nomenclaturalCode: ICZN; taxonomicStatus: accepted; **Location:** continent: Asia; islandGroup: Taiwan; island: Taiwan; country: Taiwan; countryCode: TW; stateProvince: Taipei; municipality: Da'an; locality: National Taiwan University main campus; **Identification:** identifiedBy: Hsiu-Chun Lee; dateIdentified: 2022; identificationReferences: Chen 1994, Wang 1995; **Event:** eventDate: 05/11/2021; year: 2021; month: 11; day: 5; **Record Level:** type: StillImage; language: zh; basisOfRecord: HumanObservation; source: https://www.facebook.com/groups/588350308851792/permalink/620323802321109/

#### Taxon discussion

The larva of this species was identified, based on Chen (1994) and Wang (1995).

#### Notes

Newly recognised as a host of *M.stellatus*.

### Parapolybia varia

(Fabricius, 1787)

5A4C1EFB-7495-5333-B6F8-863155B95011

https://www.gbif.org/species/1311773

https://eol.org/pages/240040

https://treatment.plazi.org/id/AD7C879B1C0AFFA95CD8FD92FC6BFCD2

#### Materials

- **Type status:** Other material. **Occurrence:** occurrenceRemarks: *Parapolybiavaria* attacked the suspended communal cocoon of *Meteorusstellatus*.; recordedBy: Ke-Hsiung Tsai; lifeStage: adult; occurrenceID: 62EFB56C-4F3A-563F-A92A-8CA576C2F0C5; **Taxon:** scientificName: *Parapolybiavaria* (Fabricius, 1787); acceptedNameUsage: *Parapolybiavaria* (Fabricius, 1787); originalNameUsage: *Vespavaria* Fabricius, 1787; kingdom: Animalia; phylum: Arthropoda; class: Insecta; order: Hymenoptera; family: Vespidae; genus: Parapolybia; specificEpithet: varia; taxonRank: species; scientificNameAuthorship: Fabricius; vernacularName: ヒメホソアシナガバチ, 變側異腹胡蜂; nomenclaturalCode: ICZN; taxonomicStatus: accepted; **Location:** continent: Asia; islandGroup: Taiwan; country: Taiwan; countryCode: TW; stateProvince: Keelung; municipality: Gangziliao trail; **Identification:** identifiedBy: Hsuan-Pu Chen, Kai-Ti Lin; dateIdentified: 2022; identificationReferences: Yamane & Wang 1996, Saito-Morooka et al. 2015; **Event:** eventDate: 01/04/2021; year: 2021; month: 4; day: 1; **Record Level:** type: StillImage; language: zh; basisOfRecord: HumanObservation; source: https://www.facebook.com/groups/393148477475231/permalink/4372047339585305/
- **Type status:** Other material. **Occurrence:** occurrenceRemarks: *Parapolybiavaria* attacked the suspended communal cocoon of *Meteorusstellatus*.; recordedBy: Su-Chuan Hung; lifeStage: adult; occurrenceID: 14CDCD61-6233-5ED6-B4A6-D36FF5591D5D; **Taxon:** scientificName: *Parapolybiavaria* (Fabricius, 1787); acceptedNameUsage: *Parapolybiavaria* (Fabricius, 1787); originalNameUsage: *Vespavaria* Fabricius, 1787; kingdom: Animalia; phylum: Arthropoda; class: Insecta; order: Hymenoptera; family: Vespidae; genus: Parapolybia; specificEpithet: varia; taxonRank: species; scientificNameAuthorship: Fabricius; vernacularName: ヒメホソアシナガバチ, 變側異腹胡蜂; nomenclaturalCode: ICZN; taxonomicStatus: accepted; **Location:** continent: Asia; islandGroup: Taiwan; island: Taiwan; country: Taiwan; countryCode: TW; stateProvince: Taipei; county: Beitou; municipality: Erziping trail, Yangmingshan National Park; **Identification:** identifiedBy: Hsuan-Pu Chen, Kai-Ti Lin; dateIdentified: 2022; identificationReferences: Yamane & Wang 1996, Saito-Morooka et al. 2015; **Event:** eventDate: 03/07/2022; year: 2022; month: 7; day: 3; **Record Level:** type: MovingImage; language: zh; basisOfRecord: HumanObservation; source: https://www.facebook.com/groups/588350308851792/permalink/775928626760625/

#### Ecology

Predation behaviour on the cocoon of *M.stellatus* by a single worker (Fig. 10) or many workers of this species (as shown in the video at https://www.youtube.com/watch?v=AYzqgeJwxOo) was reported for the first time in the present paper.

#### Taxon discussion

Although preserved specimens were unavailable and these paper wasps sometimes exhibit a wide range of colour variations (van der Vecht 1966), the available characters, based on photographs and movies, matched well with the diagnostic characters for this species listed by Yamane and Wang (1996) and Saito-Morooka et al. (2015).

### Mesochorus

33DFCB41-9D30-55D7-87E4-31C10AD48431

#### Materials

- **Type status:** Other material. **Occurrence:** occurrenceRemarks: Adult wasps emerged from cocoon of *Meteorusstellatus*.; recordedBy: Chun-Chun Deng; individualCount: 8; sex: 2 females and 6 males; lifeStage: adult; disposition: voucher NMNS; occurrenceID: F25D6EF5-6A92-5F41-823B-70A34C6548C0; **Taxon:** scientificName: *Mesochorus*; acceptedNameUsage: *Mesochorus*; kingdom: Animalia; phylum: Arthropoda; class: Insecta; order: Hymenoptera; family: Ichneumonidae; genus: Mesochorus; taxonRank: genus; nomenclaturalCode: ICZN; taxonomicStatus: accepted; **Location:** continent: Asia; islandGroup: Taiwan; island: Taiwan; country: Taiwan; countryCode: TW; stateProvince: Taipei; municipality: Da'an District; locality: Fujhoushan park; **Identification:** identifiedBy: So Shimizu, Hsuan-Pu Chen, Kai-Ti Lin; dateIdentified: 2022; identificationReferences: Araujo et al. 2018; **Event:** eventDate: 21/01/2022; year: 2022; month: 1; day: 21; **Record Level:** type: PhysicalObject; basisOfRecord: PreservedSpecimen; source: https://www.facebook.com/groups/588350308851792/permalink/665203134499842/

#### Ecology

This genus is known to be hyperparasitoids of other parasitoid wasps, including *Meteorus* species (Yu et al. 2016). Adult wasps examined in the present study emerged from the cocoon of *M.stellatus*.

#### Taxon discussion

Preserved specimens were identified as belonging to this genus, based on the key provided by Araujo et al. (2018).

## Discussion

Previous host data of *M.stellatus* suggested that its primary host consists of sphingid moth species of the genus *Macroglossum* (Fujie et al. 2021). However, our new data indicate that *M.stellatus* parasitises not only *Macroglossum* species, but also species of other genera of the tribe Macroglossini, implying polyphagous nature, as well-known and extensively studied in an extremely polyphagous koinobiont endoparasitoid *M.pulchricornis* that has more primitive lineage than *M.stellatus* of the *M.pulchricornis* clade (Fujie et al. 2021) and employs virus-like particles (VLPs) to prevent host granulocytes (Suzuki and Tanaka 2006, Suzuki et al. 2008, Suzuki et al. 2009, Yokoi et al. 2017, Maeto 2018). The geographic distribution of these hosts is wider than the currently-known range of *M.stellatus*, suggesting that *M.stellatus* is also potentially distributed in a wider area (e.g. China and the Philippines). Our data suggest that *M.stellatus* is predominantly distributed in the subtropical Oriental Region of the Far East and at least in the southern area of the Palaearctic Region. However, our interest is in their true northernmost range because one of their host species (*Ma.pyrrhosticta*) is also distributed in the subarctic Hokkaido in Japan. Although current data on *M.stellatus* are insufficient to understand the true host range, their parasitoid mechanisms and strategies and distribution, we will possibly be able to reveal their comprehensive biology through continued data collection and collaboration with citizen scientists via social media.

The pendulous communal cocoons of *M.stellatus* are occasionally attacked by the social wasp predator, *P.varia*, intensively. According to Starr (1992), the local abundance of *P.varia* in Taiwan is highly variable. Therefore, the strength of the local predation pressure of *M.stellatus* may be similarly variable depending on the local abundance of *P.varia*. As ecological data for *P.varia* is still poor and incomplete (e.g. van der Vecht (1966)), these data on the predation behaviour of *P.varia* could be important knowledge for understanding the biology of not only *M.stellatus*, but also *P.varia*.

The suspension of the cocoon by a cable has been considered to make the pupating wasp inaccessible to certain potential enemies (Shaw and Huddleston 1991, Zitani and Shaw 2002, Zitani 2003, Quicke et al. 2006, Shirai and Maeto 2009, Maeto 2018). However, intensive attacks on the suspended cocoon of *M.stellatus* by *P.varia* suggest that the suspended large cocoon may attract the attention of enemies, thereby probably increasing the risk of attack by predators with strong mandibles (or beaks) and high flight ability. In particular, large communal cocoons provide a stable foothold to relatively large-sized enemies, such as paper wasps, while small solitary cocoons do not. On the other hand, as we observed, the suspended communal cocoon was moderately to strongly swaying and spinning due to natural winds and the flapping of the paper wasps' wings, sometimes resulting in the paper wasps giving up landing and attacking it if it was unstable. These factors suggest that the function and evolution of the long cable and the communal cocoons are more complicated than previously hypothesised. Therefore, as a step towards understanding it, the relationships between the cable length and the cocoons' size and the impact of the intensive attacks by *P.varia* and other enemies, including hyperparasitoids, should be evaluated in future studies.

All data of *M.stellatus* observed in the present study were based on cocoons and the majority of it was recorded before being described as the new species. This suggests that their communal star-shaped cocoon suspended by a significantly long cable would have likely caught the interest of not only natural enemies, as suggested above, but also many citizen scientists. In contrast, relatively fewer data for adults from social media suggest that the small body size makes citizen scientists difficult to find them. In addition, the lack of uniqueness of adult morphology may not attract interest from citizen scientists. Therefore, social media posts are valuable for understanding biodiversity and natural history, but artificial biases should always be considered when we use such data.

One of the most interesting and important features of parasitoid wasps is their tremendous diversification through the evolution of interaction between the wasps and their hosts. As recently highlighted in Darwin wasps (Klopfstein et al. 2019), the number of professional scientists of parasitoid wasps is insufficient to fully reveal their biodiversity and evolutionary history. Therefore both wasps' and hosts' data are still scarce. However, much biodiversity and natural history data can now be found by the contribution of citizen scientists on social media platforms, as demonstrated in the present study. Consequently, the biodiversity and evolutionary history of parasitoid wasps may be significantly uncovered by compiling and analysing such data. Furthermore, many scientifically interesting and important data have been shared on social media by citizen scientists, but most of them have never been published in scientific publications. Therefore, professional researchers should continue to monitor social media posts and maintain positive relationships with citizen scientists and should investigate and publish scientifically valuable information found on social media to contribute to revealing biodiversity and the evolution of life on Earth.

## Supplementary Material

XML Treatment for Meteorus stellatus

XML Treatment for Cechetra minor

XML Treatment for Hippotion celerio

XML Treatment for Macroglossum passalus

XML Treatment for Macroglossum sitiene

XML Treatment for Parapolybia varia

XML Treatment for Mesochorus

## Figures and Tables

**Table 1.** A list of online citizen-science groups on Facebook focused on Taiwanese insects. These groups were utilised to compile digital occurrence data of *M.stellatus* from Taiwan. The number of group members was verified on 26 January 2023.

English name	Abbreviation	Mandarin name	Members
Ichneumonoidea of Taiwan	IchT	姬繭風—臺灣的姬蜂總科	964
Insects Forum of Taiwan	IFT	昆蟲各種問題貼圖討論區	33,488
Taiwan Hymenopterist Club	THC	臺灣膜翅目研究社	2,931

**Table 2.** Abbreviations, full names and curators for repositories.

Abbreviation	Repository name	Curator
KPM	Kagoshima Prefectural Museum, Kagoshima, Japan	Atsuko Nakamine
NMNS	National Museum of Natural Science, Taichung, Taiwan	Jing-Fu Tsai
TARI	Taiwan Agricultural Research Institute, Taichung, Taiwan	Chi-Feng Lee

**Table 3.** List of sources for digital occurrence data of *Meteorusstellatus* Fujie, Shimizu & Maeto, 2021. The IDs link to the identifiers in the "Materials" section of *M.stellatus*.

ID	Locality	Adult	Cocoon	Host	Parasitoid	Predator
a	TW	n/a	Fig. 8a	Fig. 8b,c	n/a	n/a
b	TW	n/a	Fig. 4e	n/a	n/a	n/a
c	TW	n/a	Fig. 4h	n/a	n/a	n/a
d	TW	n/a	Fig. 9e	n/a	Fig. 9e	n/a
e	TW	n/a	Fig. 9a	n/a	Fig. 9a	n/a
f	TW	n/a	Fig. 4b	n/a	n/a	n/a
g	TW	n/a	Fig. 4a	n/a	n/a	n/a
h	TW	n/a	Fig. 4i-k	n/a	n/a	n/a
i	TW	n/a	Fig. 4f,g	n/a	n/a	n/a
j	TW	n/a	Fig. 4c	n/a	n/a	n/a
k	TW	n/a	Fig. 10	n/a	n/a	Fig. 10
l	TW	Fig. 2	Fig. 6	Fig. 6d	n/a	n/a
m	TW	n/a	Fig. 9c,d	n/a	Fig. 9c,d	n/a
n	TW	n/a	Fig. 5b	Fig. 5a	n/a	n/a
o	TW	n/a	Youtube	n/a	n/a	Youtube
p	TW	n/a	Fig. 4d	n/a	n/a	n/a
q	JP	Fig. 11b,c	Fig. 11a,b	n/a	n/a	n/a
r	TW	Fig. 7d	Fig. 7a-d	Fig. 7a	n/a	n/a
s	TW	n/a	Fig. 9b	n/a	Fig. 9b	n/a
t	TW	n/a	Fig. 7e	Fig. 7e	n/a	n/a
u	TW	n/a	Fig. 9f	n/a	Fig. 9g,f	n/a
v	TW	Fig. 3b	Fig. 3a	n/a	n/a	n/a

**Table 4.** List of sources and depositories for physical occurrence data of *Meteorusstellatus* Fujie, Shimizu & Maeto, 2021. The IDs link to the identifiers in the "Materials" section of *M.stellatus*.

ID	Locality	Adult	Cocoon	Host	Parasitoid	Predator
w	TW	n/a	NMNS	n/a	n/a	n/a
x	TW	n/a	NMNS	n/a	n/a	n/a
y	TW	TARI	n/a	n/a	n/a	n/a
z	TW	TARI; Fig. 12a	n/a	n/a	n/a	n/a
aa	TW	n/a	TARI; Fig. 12c	n/a	n/a	n/a
ab	TW	TARI; Fig. 2	n/a	n/a	n/a	n/a
ac	JP	KPM	KPM	n/a	n/a	n/a

**Table 5.** Insects associated with *Meteorusstellatus* Fujie, Shimizu & Maeto, 2021. "Lep." = "Lepidoptera", "Hym." = "Hymenoptera".

Type	Order	Family	Species	Source
Host	Lep.	Sphingidae	*Cechetraminor*	Fig. 8b,c
Host	Lep.	Sphingidae	*Hippotioncelerio*	Fig. 5a
Host	Lep.	Sphingidae	*Macroglossumpassalus*	Fujie et al. (2021); Fig. 7a,e
Host	Lep.	Sphingidae	*Macroglossumpyrrhosticta*	Fujie et al. (2021)
Host	Lep.	Sphingidae	*Macroglossumsitiene*	Fig. 6d
Parasitoid	Hym.	Ceraphronidae	*Aphanogmus* sp.	Fujie et al. (2021)
Parasitoid	Hym.	Eulophidae	*Tetrastichus* sp.	Fujie et al. (2021)
Parasitoid	Hym.	Eurytomidae	*Eurytoma* sp.	Fujie et al. (2021)
Parasitoid	Hym.	Ichneumonidae	*Mesochorus* sp.	Fig. 9f,g
Parasitoid	Hym.	Pteromalidae	n/a	Fig. 9a-d
Parasitoid	Hym.	Trichogrammatidae	n/a	Fig. 9e
Predator	Hym.	Vespidae	*Parapolybiavaria*	Fig. 10; Youtube
